# The Drosophila tumor necrosis factor receptor, Wengen, couples energy expenditure with gut immunity

**DOI:** 10.1126/sciadv.add4977

**Published:** 2023-06-09

**Authors:** Rihab Loudhaief, Rouba Jneid, Christian Fokdal Christensen, Duncan J. Mackay, Ditte S. Andersen, Julien Colombani

**Affiliations:** Department of Biology, Faculty of Science, University of Copenhagen, Universitetsparken 15, Build. 3, 3rd floor, room 439, 2100 Copenhagen O, Denmark.

## Abstract

It is well established that tumor necrosis factor (TNF) plays an instrumental role in orchestrating the metabolic disorders associated with late stages of cancers. However, it is not clear whether TNF/TNF receptor (TNFR) signaling controls energy homeostasis in healthy individuals. Here, we show that the highly conserved Drosophila TNFR, Wengen (Wgn), is required in the enterocytes (ECs) of the adult gut to restrict lipid catabolism, suppress immune activity, and maintain tissue homeostasis. Wgn limits autophagy-dependent lipolysis by restricting cytoplasmic levels of the TNFR effector, TNFR-associated factor 3 (dTRAF3), while it suppresses immune processes through inhibition of the dTAK1/TAK1-Relish/NF-κB pathway in a dTRAF2-dependent manner. Knocking down dTRAF3 or overexpressing dTRAF2 is sufficient to suppress infection-induced lipid depletion and immune activation, respectively, showing that Wgn/TNFR functions as an intersection between metabolism and immunity allowing pathogen-induced metabolic reprogramming to fuel the energetically costly task of combatting an infection.

## Teaser

The Drosophila TNF receptor, Wengen, controls immunometabolism and promotes tissue homeostasis in healthy guts.

## INTRODUCTION

The family of tumor necrosis factors (TNFs) participates in diverse processes ranging from cell proliferation, differentiation, and apoptosis to innate and adaptive immunity. While TNFs are important regulators of tissue homeostasis and immunity, they are also, by virtue of their proinflammatory properties, potent drivers of tumorigenesis and inflammatory diseases (*1*). In addition to their well described proinflammatory functions, TNFs are thought to play a key role in orchestrating the metabolic rewiring associated with disease conditions in both flies and mammals (*1*, *2*). While this suggests that TNF can act as a metabolic hormone, it is not known whether TNF–TNF receptor (TNFR) signaling regulates metabolic processes in healthy individuals. A recent study in flies showed that ectopic activation of Drosophila tumor necrosis factor receptor–associated factor 3 (dTRAF3), precursor of TRAFs 1, 2, 3, and 5 in mammals and immediate downstream effector of TNFRs, can alter lipid metabolism (*3*). Although this study reported on the effects of ectopic expression of dTRAF3 (*3*), it opens the possibility that TNF/TNFR signaling might also regulate energy expenditure/storage in adult immune tissues, such as the gut.

The mammalian TNF/TNFR signaling network is complex with 19 ligands and 29 interacting receptors belonging to the TNF and TNFR superfamilies (*4*). Because of the prominent role of TNF-α in inflammatory diseases, such as inflammatory bowel disease (IBD), and consequently its potential as a drug target, TNF-α and its downstream effectors have been particularly well characterized. Binding of TNF-α to TNFR1 results in the formation of complex I, which consists of TNFR1-associated death domain protein, TRAF2, and receptor-interacting serine/threonine kinase 1 (RIP1). Subsequently, TRAF2 recruits and activates transforming growth factor β–activated kinase 1 (TAK1) triggering c-JUN N-terminal kinase (JNK) and nuclear factor κB (NF-κB) activation to induce immunity and promote survival [reviewed in (*4*)]. While the role of TNF-α/TNFR1 signaling in innate/adaptive immunity and tissue homeostasis is well established, its potential function in regulating energy homeostasis and how this is coupled with immune processes has not been explored.

In contrast to the mammalian TNF-TNFR network, Drosophila has a much simpler TNF ligand–receptor system composed of a unique TNF ligand, Eiger (Egr), and two TNFRs, Grindelwald (Grnd) and Wengen (Wgn) (*5*–*8*); hence providing a convenient model for studying TNF-TNFR–mediated processes. While TNFs regulate JNK/mitogen-activated protein kinase (MAPK) signaling in both flies and mammals, ectopic activation of Egr is not sufficient to trigger immune responses in flies (*6*). Therefore, the fly immune deficiency (IMD) pathway, which is activated upon binding of bacterially derived peptidoglycans (PGNs) to PGN recognition proteins (PGRPs), is considered to be the sole regulator of dTAK1–NF-κB/Relish–mediated immune responses (*9*). Nevertheless, IMD shares structural similarities with mammalian RIP1, and the downstream components of the IMD pathway, including dTAK1, are homologous to the signaling cassette downstream of TRAF2 in the TNFR1 pathway (fig. S1A) (*9*), raising the possibility that Relish/NF-κB signaling could be regulated in a TNF/TNFR-TRAF–dependent and PGN/PGRP-independent manner. Consistent with this, ectopic expression of dTRAF2 triggers NF-κB/Relish signaling in the larval fat body (*10*). However, as this study relied on ectopic expression of dTRAF2 (*10*), it remains unclear how and whether dTRAF2 signaling controls immunity in physiological conditions.

Here, we show that the fly TNFR, Wgn, plays a key role in coupling energy expenditure with immunity in the adult fly gut. In homeostatic conditions, Wgn restricts cytoplasmic dTRAF3 levels in gut enterocytes (ECs) to suppress lipid catabolism and maintain tissue homeostasis, while it suppresses immunity through the highly conserved dTAK1/TAK1–Relish/NK-κB pathway in an dTRAF2-dependent, but dTRAF3-independent, manner. While Wgn suppresses dTRAF3-mediated lipolysis independently of its ligand, Egr/TNF, our data suggest that Wgn-dTRAF2–mediated suppression of immunity occurs in an Egr-dependent manner. Mimicking Wgn gain of function by knocking down dTRAF3 or overexpressing dTRAF2 is sufficient to suppress infection-induced lipid depletion and immune activation, respectively. These observations show that Wgn/TNFR functions as metabolic switch reprogramming metabolism toward energy expenditure in conditions associated with higher levels of immune activity while suppressing lipid catabolism and inflammation in healthy guts.

## RESULTS

### Wgn/TNFR restricts lipid catabolism independently of its ligand, Egr/TNF, in the gut

**Fig. 1.**
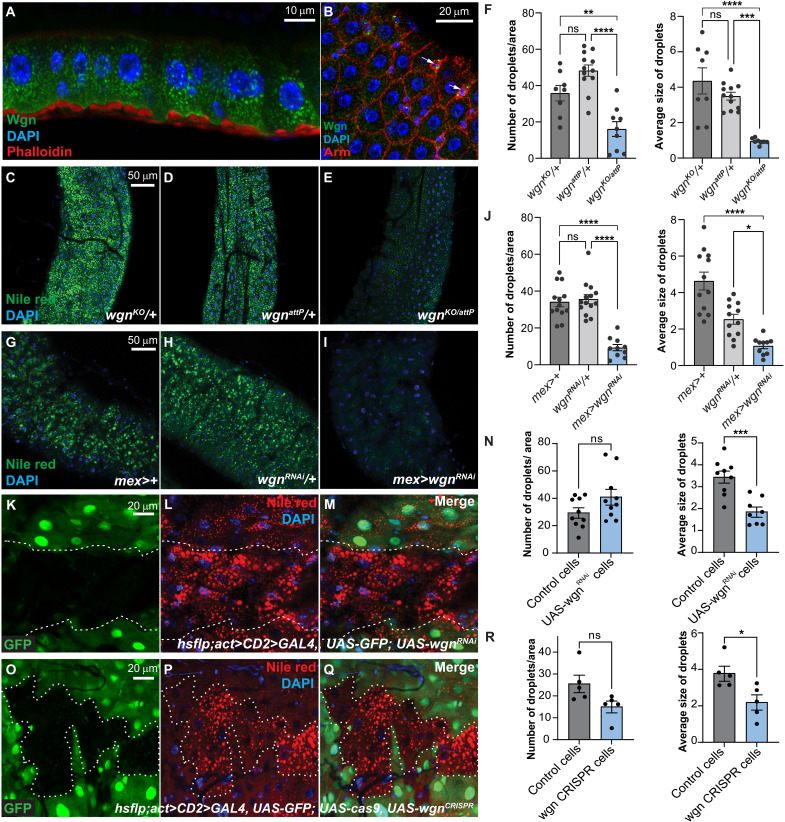
EC-specific knockdown of Wgn in the adult gut triggers lipid catabolism. (**A** and **B**) Dissected guts stained for Wgn (green), Phalloidin [(A), red], or Armadillo [(B), Arm, red] and 4′,6-diamidino-2-phenylindole (DAPI; blue) showing that Wgn is localized in intracellular vesicles in ECs (A) and Arm-rich progenitor cells (B, white arrows). (**C** to **J**) The R2 region of guts dissected from control (C), (D), (G) and (H), *wgn* mutant animals (E), or animals with EC-specific knockdown of Wgn (I) and stained with the lipid dye Nile Red (green). The number and size of lipid droplets in (C) to (E) and (G) to (I) were quantified using FIJI software (F) and (J). (**K** to **R**) The R2 region of dissected guts carrying mosaics of wildtype (non–GFP-labeled) and Wgn depleted (GFP-labeled) cells stained for Nile Red (red). Wgn knockdown using RNAi (K) to (M) or CRISPR-Cas9 (O) to (M) triggers depletion of lipids in an autonomous fashion. The number and size of lipid droplets in (K) to (M) and (O) to (Q) were quantified for each condition (J) and (N). Error bars represent SEM; *P < 0.05, **P < 0.01, ***P < 0.001, and ****P < 0.0001.

To investigate how TNF-TNFR signaling affects energy homeostasis in the gut, we first examined the expression pattern of the two fly TNFRs, Wgn and Grnd, in the adult gut. While we could not detect any Grnd protein, Wgn was present in intracellular vesicles in ECs, progenitor cells (Fig. 1B, white arrows), and the visceral muscles (VMs) surrounding the gut (Fig. 1, A and B, and fig. S1, B to E). The observation that the majority of Wgn is localized in intracellular vesicles rather than at the plasma membrane is consistent with our previous observations in larval epithelia and a recent study in the embryonic tracheal system (*11*, *12*). To test whether TNFR signaling regulates energy homeostasis in homeostatic conditions, we quantified lipid levels in *wgn* and *grnd* null mutant guts. Notably, *wgn*, but not *grnd*, mutant guts were depleted of lipids, suggesting an important homeostatic function of Wgn in regulating lipid metabolism (Fig. 1, C to E, and fig. S1, F to J). Notably, this requirement for Wgn did not depend on the unique fly TNF, Egr, as lipid stores in *egr* null mutant guts were unaffected (fig. S1, K to M). We subsequently used RNA interference (RNAi) to knock down Wgn in VMs, where it is highly expressed (fig. S1C), and ECs and found that Wgn is specifically required in ECs to maintain normal levels of lipids (Fig. 1, G to J, and fig. S1, N to S). Similarly, knockdown of *wgn* in clones using RNAi or CRISPR-Cas9–mediated depletion caused an autonomous reduction of lipids in the mutant tissue (Fig. 1, K to R). Together, our data show that Wgn plays a key role in maintaining lipid homeostasis in the adult gut, a function that does not depend on its ligand Egr/TNF.

### Depletion of Wgn in ECs triggers autophagy-dependent lipolysis and sensitivity to starvation

**Fig. 2.**
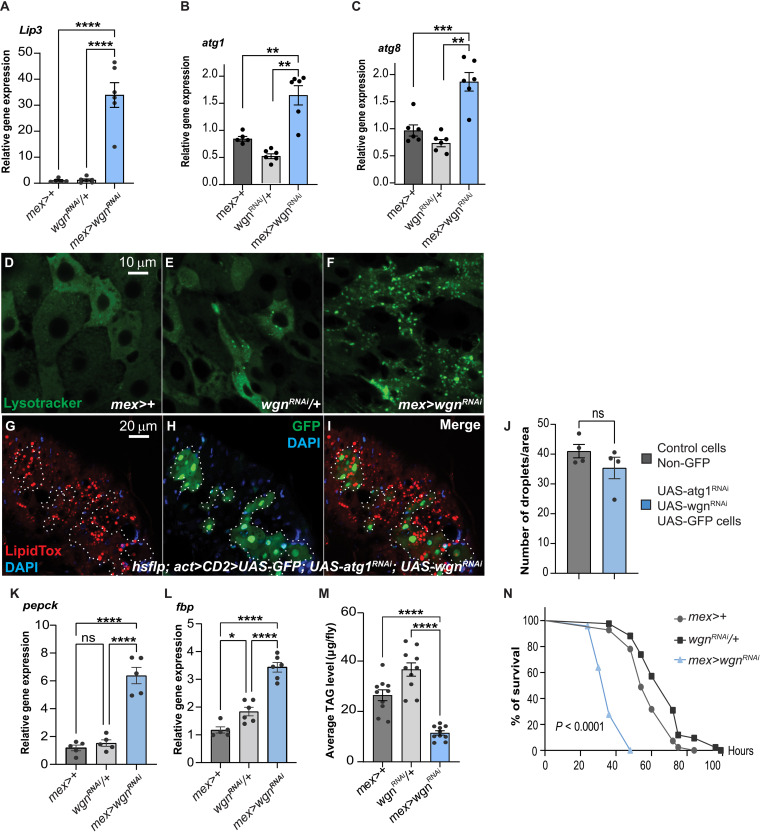
EC-specific knockdown of Wgn triggers autophagy-dependent lipolysis and sensitivity to starvation. (**A** to **C**) qPCR analyses on dissected guts showing an up-regulation of Lip3 and genes required for autophagosome formation in guts with EC-specific Wgn knockdown. (**D** to **F**) Dissected guts from adult control animals (D) and (E) and animals with EC-specific Wgn depletion (F) stained with lysotracker (in green) to label acidic compartments including autolysosomes in the R4 region. (**G** to **J**) Adult guts carrying mosaics of wildtype (non–GFP-labeled) and Wgn + Atg1 depleted (GFP-labeled) cells stained with the lipid dye LipidTox (in red) in the R5 region. Simultaneous knockdown of Wgn and Atg1 rescues the lipid catabolism observed upon knockdown of Wgn alone as quantified in (J). (**K** and **L**) qPCR analyses on dissected guts showing an up-regulation of the gluconeogenic enzymes, phosphoenolpyruvate carboxy kinase (pepck) (K), and fructose 1,6-bisphosphate (fbp) (L), in guts with EC-specific Wgn knockdown. (**M**) Quantifiation of fat body TAG levels in control animals and animals with EC-specific knockdown of Wgn. (**N**) Reduced resistance of flies with EC-specific Wgn depletion (blue) to starvation compared with control animals (gray). Error bars represent SEM; *P < 0.05, **P < 0.01, ***P < 0.001, and ****P < 0.0001.

To better understand what triggered the lipid depletion, we tested the effect of EC-specific Wgn knockdown on feeding behavior and enzymes involved in lipogenesis and lipolysis. Knockdown of Wgn increased food intake and the expression of FAS and ACC, two key enzymes of de novo lipogenesis, showing that the reduction in gut lipids is not caused by reduced food intake or impaired lipogenesis (fig. S2, A to G). By contrast, Wgn depletion strongly induced lipase 3 (Lip3), suggesting that lipid catabolism underpin lipid depletion in this condition (Fig. 2A). Consistent with this, we observed an increase in the expression of autophagy genes and autophagosomes labeled by lysotracker, a dye labeling acidic organelles including autolysosomes, suggesting that Wgn knockdown triggers autophagy-mediated lipolysis (Fig. 2, B to F). To confirm this, we simultaneously knocked down Wgn and the facultative autophagy protein, Atg1, and found that this was sufficient to rescue the lipid depletion observed upon EC-specific Wgn knockdown (Fig. 2, G to J). Furthermore, we observed an induction of the starvation-responsive gluconeogenic enzymes, Pepck and Fbp, in Wgn depleted guts (Fig. 2, K to L), suggesting that Wgn loss of function triggers a starvation-like response.

We next investigated the global effects of knocking down Wgn in ECs by measuring fat body and whole-body triacylglycerol (TAG) levels. While EC-specific Wgn depletion significantly decreased fat body and global TAG levels (Fig. 2M and fig. S2H), it did not alter the expression of enzymes involved in lipogenesis or lipid mobilization in non-intestinal tissues (fig. S2, I to N), showing that the decrease in global TAG levels is caused by reduced translocation of lipids from the gut to other tissues. Consistent with a global reduction in TAG levels, flies with EC-specific knockdown of Wgn and *wgn* null mutant animals both displayed increased sensitivity to starvation (Fig. 2, M and N, and fig. S2, O and P). Together these data show that Wgn plays a key role in maintaining energy homeostasis in the adult gut.

### Wgn restricts cytoplasmic pools of dTRAF3 and dTRAF3-mediated lipid catabolism

**Fig. 3.**
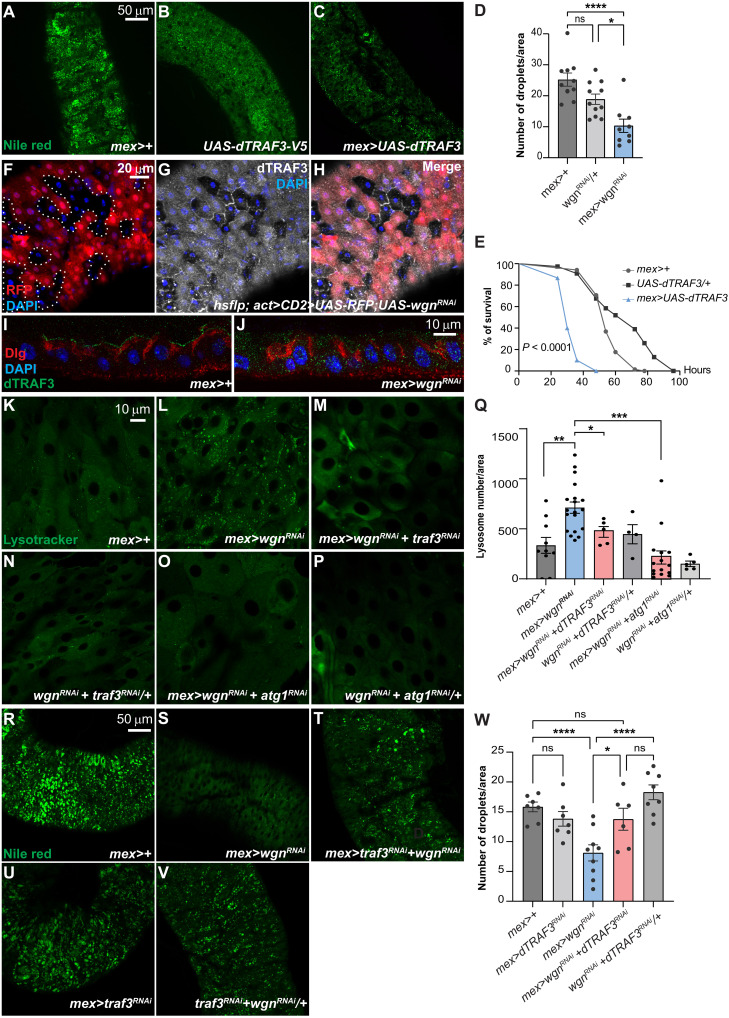
Wgn restricts cytoplasmic pools of dTRAF3 and dTRAF3-dependent lipid catabolism. (**A** to **C**) The R2 region of adult guts dissected from control animals (A) and (B) and animals with EC-specific ectopic expression of TRAF3 (C) stained with the lipid dye Nile Red (in green). (**D**) Number of lipid droplets per area was quantified in each of the conditions in (A) to (C). (**E**) Reduced resistance of flies with EC-specific ectopic dTRAF3 expression (blue) to starvation compared with control animals (gray). (**F** to **H**) Adult guts carrying mosaics of wild-type (non–RFP-labeled) and Wgn depleted (RFP-labeled) cells and stained for dTRAF3 (white). Knockdown of Wgn results in the loss of membrane-associated and accumulation of cytoplasmic dTRAF3 in the R5 region. (**I** and **J**) Transversal section of guts dissected from control animals (I) and animals with EC-specific Wgn depletion (J) and stained for dTRAF3 (green) and Disc-large (Dlg, red) showing that dTRAF3 localizes to the membrane in control guts, while it accumulates in intracellular vesicles in Wgn-depleted guts. (**K** to **P**) The R5 region of dissected guts from adult control animals (K), (N), and (P) and animals with EC-specific depletion of Wgn alone (L), Wgn and dTRAF3 (M), or Wgn and Atg1 (O) stained with lysotracker (in green) to label acidic compartments including autolysosomes. (**Q**) Quantification of number of lysotracker-positive vesicles per area for each of the conditions in (K) to (P). (**R** to **V**) The R4-R5 region of adult guts dissected from control animals (R) and (V) and animals with EC-specific depletion of Wgn (S), dTRAF3 (U), or Wgn and dTRAF3 (T) stained with the lipid dye Nile Red (in green). (**W**) Quantification of number of lipid droplets per area for each condition in (R) to (V) shows that dTRAF3 is required for the lipid catabolism triggered by EC-specific Wgn knockdown. Error bars represent SEM; *P < 0.05, **P < 0.01, ***P < 0.001, and ****P < 0.0001.

To identify the downstream effectors responsible for the lipid depletion observed in response to EC-specific knockdown of Wgn, we manipulated the activity of all three fly dTRAFs (dTRAF1 to dTRAF3) and analyzed the effect on lipids. While individual knockdown of dTRAFs 1, 2, and 3 did not affect lipid homeostasis (Fig. 3W and fig. S3, A to F), ectopic expression of dTRAF3 (Fig. 3, A to D), but not dTRAF1 and dTRAF2 (fig. S3, G to N), caused lipid depletion and rendered flies sensitive to starvation (Fig. 3E). Therefore, we next investigated the effect that knockdown of Wgn has on dTRAF3 protein levels and cellular localization using our anti-dTRAF3 (see Materials and Methods) or an anti-V5 antibody to visualize endogenous dTRAF3 and V5-tagged dTRAF3, respectively. Notably, while dTRAF3 mainly localized to the membrane in control tissues {Fig. 3, F to H [non–red fluorescent protein (RFP)–labeled cells], and I, and fig. S3, O to R}, knockdown of Wgn led to a substantial decrease in membrane-associated dTRAF3 accompanied by an increase in of dTRAF3 in intracellular vesicles [Fig. 3, F to H (RFP labeled cells), and I to J]. This suggests that Wgn is required to restrict cytoplasmic pools of dTRAF3 and that dTRAF3 might mediate the effect on lipid metabolism induced by Wgn knockdown. The simultaneous knockdown of Wgn and dTRAF3 reduced the induction of *lip3* and genes required for autophagosome formation and suppressed the autophagic response and lipid depletion triggered by Wgn loss of function (Fig. 3, K to W, and fig. S3, S and T). Notably, and in accordance with previous reports, we observed a similar increase in cytoplasmic dTRAF3 levels following knockdown of the E3 ubiquitin ligase, NOPO [TNFR-interacting protein (TRIP) in mammals] (fig. S3, U to W) (*3*), suggesting that Wgn might promote NOPO-dependent degradation of cytoplasmic dTRAF3 to restrict autophagy-mediated lipolysis. Ectopic expression of dTRAF3 was previously reported to promote the formation of Cad99c^+^dTRAF3^+^ vesicles and reactive oxygen species (ROS) production (*3*). We did not observe spontaneous Cad99c^+^ vesicles formation or elevated ROS production upon EC-specific or global Wgn knockdown (fig. S3X), and hence, the dTRAF3-mediated lipolysis, associated with Wgn loss of function, is likely to be independent of dTRAF3’s function in Cad99c^+^dTRAF3^+^ vesicles to induce the Dual oxidase (DUOX)-dependent ROS production.

### Wgn/dTRAF2 suppresses dTAK1/TAK1-Relish/NF-𝛋B–mediated immunity in homeostatic conditions

**Fig. 4.**
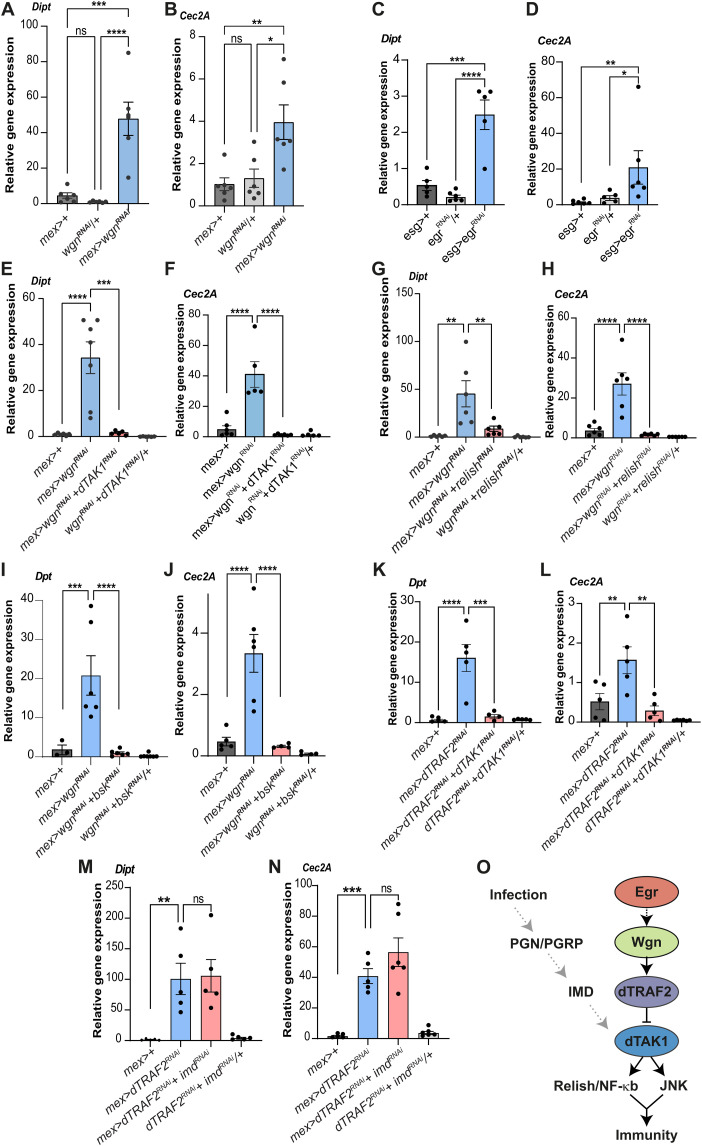
Wgn and dTRAF2 restrict Relish/NF-kB- and JNK-mediated immunity. (**A** and **B**) qPCR analyses on dissected adult guts show up-regulation of the AMPs, Diptericin (Dipt), and Cecropin 2A (Cec2A) in guts with EC-specific Wgn knockdown (blue bars). (**C** and **D**) qPCR analyses on dissected adult guts showing up-regulation of Dipt and Cec2A in guts with knockdown of Egr in progenitor cells. (**E** to **J**) qPCR analyses on dissected adult guts show up-regulation of Dipt and Cec2A in guts with EC-specific Wgn knockdown, which is suppressed upon simultaneous knockdown of Wgn and dTAK1 (E) and (F), Wgn and Relish (G) and (H), or Wgn and Bsk/JNK (I) and (J). (**K** and **L**) qPCR analyses on dissected adult guts showing up-regulation of Dipt and Cec2A upon EC-specific dTRAF2 knockdown, which is suppressed upon simultaneous knockdown of dTRAF2 and dTAK1. (**M** and **N**) qPCR analyses on dissected adult guts showing up-regulation of Dipt and Cec2A upon EC-specific dTRAF2 knockdown, which is not suppressed upon simultaneous knockdown of dTRAF2 and IMD. (**O**) Model depicting the Wgn/dTRAF2-dependent suppression of Relish- and JNK-mediated immunity in homeostatic conditions (black arrows) and the PGN/PGRP-IMD–dependent regulation of immunity, which is turned off in homeostatic conditions (gray arrows) but triggered by infections.

In flies, the PGN/PGRP-dependent activation of the IMD pathway plays a critical role in promoting immunity and resistance to enteric infections. While PGNs are thought to be the sole regulators of NF-κB/Relish–mediated immunity, the notable similarity between the fly IMD and mammalian TNFR1 pathways raises the possibility that Egr/TNF-Wgn/TNFR signaling might also regulate NF-κB/Relish–mediated immune processes (fig. S1A). The expression of the antimicrobial peptides (AMPs), Diptericin (Dipt) and Cecropin 2A (Cec2A), both transcriptional targets of Relish/NF-κB, were elevated in *wgn* null mutant guts and upon EC-specific knockdown of *wgn* (Fig. 4, A and B, and fig. S4, A and B). Notably, we observed a similar strong induction of AMPs in *egr* mutant flies, suggesting that Egr and Wgn are both required to suppress immunity in homeostatic conditions (fig. S4, C and D). Previous studies reported that Egr is expressed in ECs in the most anterior (R1) and posterior part (R5) of the gut, while it is strongly expressed in progenitor cells all along the gut, where it is required to promote the proliferative response to infection (*13*, *14*). Knockdown of Egr in ECs and progenitor cells showed that Egr is required in progenitor cells, but not ECs, to restrict immunity (Fig. 4, C and D, and fig. S4, E and F). Depletion of Wgn in progenitor cells moderately increased Dipt levels, while it had no effect on Cec2A expression (fig. S4, G and H), suggesting intestinal stem cells (ISC) and enteroblasts (EB)–derived Egr signals both autonomously and non-autonomously in ECs to restrict immunity. Consistent with a conserved role of TNFR signaling in regulating dTAK1–NF-κB–mediated immunity, simultaneous knockdown of Wgn and either dTAK1 or Relish/NF-κB completely suppressed the induction of AMPs associated with Wgn loss of function (Fig. 4, E to H). EC-specific knockdown of Basket (Bsk)/JNK also suppressed AMP expression (Fig. 4, I and J), showing that Bsk/JNK and Relish/NF-κB are both needed to trigger the immune response associated with Wgn loss of function. This is consistent with a previous report showing that JNK and Relish are required in a nonredundant manner for the induction of AMPs in the larval fat body (*15*). Notably, the immune-regulatory function of Wgn was not mediated through an effect on dTRAF3 (fig. S4I). This prompted us to investigate whether manipulating levels of other TRAFs might affect immunity. Depletion of dTRAF2, but not dTRAF1, strongly induced AMP expression (fig. S4, J and K), suggesting that Wgn uses its canonical partner, dTRAF2, to suppress Relish/NK-κB–mediated immunity. Consistent with this, the induction of immunity caused by EC-specific dTRAF2 depletion was efficiently suppressed upon knockdown of dTAK1 (Fig. 4, K and L). To better understand the relationship between the PGN/RGRP-IMD– and Egr/Wgn-dTRAF2–dependent regulations of immunity, we investigated whether dTRAF2 might prevent IMD-dependent activation of the dTAK1/TAK1-Relish/NF-κB pathway in homeostatic conditions. The induction of AMP expression triggered by dTRAF2 inactivation was not suppressed by IMD knockdown (Fig. 4, M and N), showing that PGN/PGRP signaling is dispensable for dTAK1 activation in homeostatic conditions. Together this suggests that Egr-Wgn–dependent regulations suppress immunity in homeostatic conditions, while PGN/PGRP signaling promotes infection-induced immune processes (Fig. 4O). The lipid catabolism associated with Wgn loss of function is not caused by excess immune activity, as simultaneous knockdown of dTAK1, which efficiently suppresses the induction of AMPs, does not prevent lipid depletion (fig. S4, K to S). Overall, our results show that in homeostatic conditions, Wgn inhibits dTRAF3-dependent lipolysis, while it restricts immunity in a dTRAF3-independent and dTRAF2-dependent manner.

### Wgn restricts dTRAF3/dTAK1-JNK signaling to maintain gut homeostasis

**Fig. 5.**
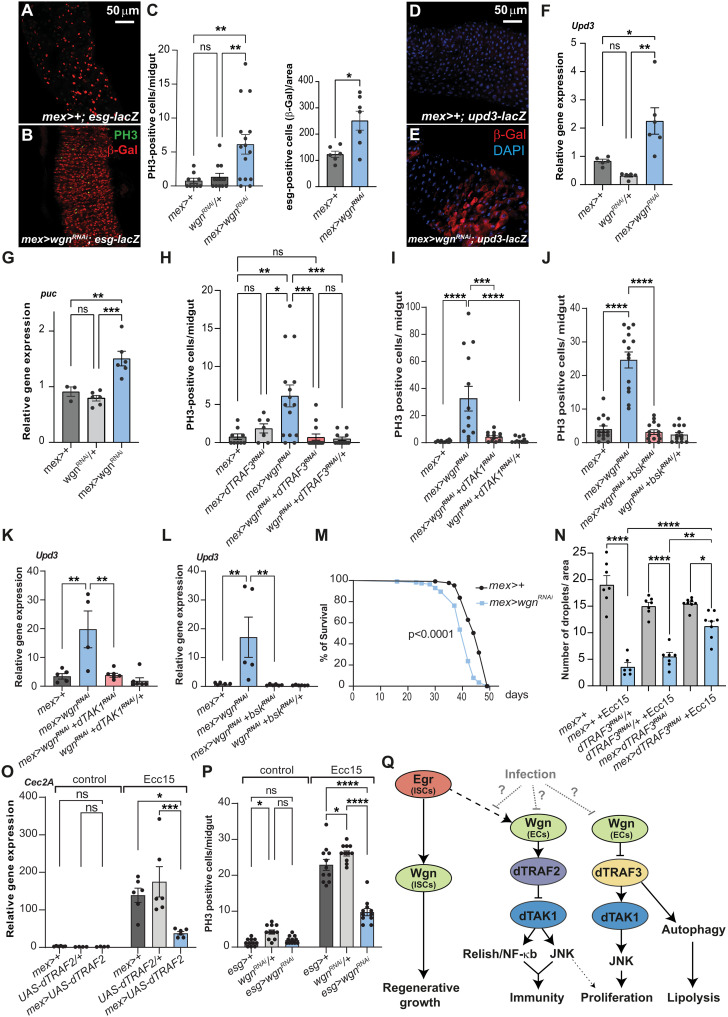
Wgn is required in ECs to maintain tissue homeostasis and in ISCs for regenerative growth. (**A** to **C**) The number of phospho-histone 3 (PH3)–positive cells (green) and progenitor cells (red) was counted in adult guts dissected from control animals (A) or animals with EC-specific Wgn knockdown (B) in R4-R5. (**D** to **G**) EC-specific knockdown of Wgn increases the expression of unpaired 3 [upd3-lacZ; (D) to (F)] and the JNK target puckered [puc, (G)]. (**H** to **J**) The increase in PH3-positive cells caused by EC-specific Wgn knockdown is suppressed by simultaneous knockdown of Wgn and dTRAF3 (H), Wgn and dTAK1 (I), or Wgn and Bsk (J). (**K** and **L**) The increase in upd3 expression triggered by EC-specific Wgn knockdown is suppressed by simultaneous knockdown of Wgn and dTAK1 (K) or Wgn and Bsk (L). (**M**) EC-specific Wgn depletion shortens lifespan. (**N**) Quantification of number of lipid droplet per area in guts dissected from infected (blue bars) and noninfected (gray bars) control animals and animals with EC-specific dTRAF3 knockdown. (**O**) qPCR analyses on infected and noninfected guts dissected from control animals and animals with EC-specific dTRAF2 overexpression. (**P**) Knockdown of Wgn in progenitor cells suppresses the increase in PH3-positive cells triggered by 16 hours of Ecc15 infection. (**Q**) Schematic showing how Wgn control lipid metabolism and tissue homeostasis through suppression of dTRAF3 activity and immunity through JNK/Bsk and Relish/NF-κB. In response to infection, Wgn-dependent dTRAF3 inhibition and dTRAF2 activation is suppressed to promote lipid catabolism and immunity. Wgn is required in ISCs to promote infection-induced regenerative growth. Error bars represent SEM; *P < 0.05, **P < 0.01, ***P < 0.001, and ****P < 0.0001.

As mammalian TNFRs are key regulators of tissue repair and immunity, we also examined the effect of EC-specific Wgn knockdown on gut epithelial turnover. In the adult fly gut, the only proliferating cells are ISCs. Their activity is tightly controlled by multiple niche-derived signals, which restrict ISC proliferation in homeostatic conditions or stimulate growth during tissue repair (*16*–*19*). We found that ISC proliferation was significantly increased in *wgn* null mutant animals compared with control animals (fig. S5A) and upon EC-specific knockdown of Wgn in homeostatic conditions (Fig. 5, A to C). During tissue repair, JNK signaling is activated in damaged ECs resulting in the production and release of the proinflammatory cytokine, Unpaired 3 (Upd3; interleukin-6 in mammals), which acts non-autonomously to stimulate ISC proliferation. Consistent with active JNK signaling, we observed an increase in *puckered* (*puc*), a transcriptional target of the JNK pathway, and Upd3 expression in the ECs of Wgn depleted guts (Fig. 5, D to G). To investigate whether the increase in ISC proliferation associated with EC-specific Wgn knockdown is triggered by dTRAF3, we simultaneously knocked down Wgn and dTRAF3 and found that this was sufficient to suppress the proliferative response associated with EC-specific Wgn depletion (Fig. 5H and fig. S5B). This dTRAF3-dependent effect on proliferation is separate from its effect on autophagy, as simultaneous knockdown of Wgn and Atg1 did not suppress the proliferation in this condition (fig. S5, B and C). As *puc* and *upd3* are targets of JNK/Bsk, we next tested whether dTAK1-Bsk/JNK signaling mediates the proliferation associated with Wgn loss of function. EC-specific knockdown of either dTAK1 or Bsk/JNK efficiently suppressed the increase in *upd3* expression and proliferation triggered by Wgn knockdown (Fig. 5, I to L). Although dTRAF2 loss of function also triggers dTAK1-Bsk/JNK signaling and a moderate increase in proliferation (fig. S5, D and E), knockdown of dTRAF3 efficiently suppressed the proliferation associated with Wgn depletion (Fig. 5H and fig. S5B), suggesting that JNK-dependent effects on proliferation is mediated downstream of dTRAF3. Consistent with this, ectopic expression of dTRAF3 in ECs is sufficient to induce the expression of the JNK targets, *puc* and *upd3*, and increase the mitotic index (fig. S5, F to H). In agreement with an Egr-independent role of Wgn in restricting dTRAF3 activity, *egr* mutant animals did not display excess proliferation (fig. S5I).

### Depleting dTRAF3 or overexpressing dTRAF2 to mimic Wgn gain of function suppresses infection-induced lipolysis and immunity

Accelerated epithelial turnover and chronic inflammation of the gut are hallmarks of aging; therefore, we tested the effect of EC-specific knockdown of *wgn* on longevity. Consistent with a role of *wgn* in promoting gut health, we found that *wgn* mutant animals were shorter-lived (Fig. 5M and fig. S5J). This prompts the question, in which physiological context might coordination of energy metabolism, immunity, and tissue renewal be relevant? During pathogenic infections, metabolic reprogramming toward energy expenditure is thought to fuel the augmented level of immune activity required to combat the infection (*20*), suggesting that Wgn-mediated signaling might be diminished in this condition. Like Wgn loss of function, oral infection induced the expression of *lip3* and autophagy genes and triggered lipid depletion (Fig. 5N and fig. S5L). To investigate whether a decrease in Wgn activity is required for the induction of immunity and lipolysis associated with infections, we mimicked Wgn gain of function by depleting dTRAF3 or overexpressing dTRAF2. Notably, EC-specific dTRAF3 depletion was sufficient to suppress infection-induced lipid depletion (Fig. 5N), suggesting that Wgn-mediated repression of dTRAF3 activity is prevented in this condition. Similarly, overexpression of dTRAF2 was sufficient to suppress the induction of AMPs associated with oral infection (Fig. 5O), in agreement with reduced Wgn-dTRAF2 signaling in this condition. By contrast, EC-specific dTRAF3 knockdown did not suppress the proliferative response triggered by infections, showing that dTRAF3 is not required in ECs to promote regenerative growth (fig. S5K). Previous studies have shown that Egr is required in progenitor cells to promote their divisions in response to an infection, and we therefore tested whether Wgn is required in these cells to support regeneration (*14*). Knockdown of either Wgn or Egr in progenitor cells was sufficient to reduce the proliferative response triggered by oral infection (Fig. 5P and fig. S5M), identifying a Egr/Wgn-dependent role in tissue repair. In summary, our data show that Wgn/TNFR functions in ECs as a metabolic switch reprogramming metabolism toward energy expenditure in conditions associated with higher levels of immune activity while restricting lipid catabolism and immunity in homeostatic conditions (Fig. 5Q). In addition, Egr and Wgn are required in progenitor cells to promote tissue repair (Fig. 5Q). Although we found that oral infection with the Gram-negative pathogen *Erwinia carotovora carotovora* 15 (*Ecc15*) significantly decreased *wgn* expression levels in the gut (fig. S5N), overexpression of Wgn was not sufficient to suppress infection-induced lipolysis and AMP expression (fig. S5, O and P), suggesting that Wgn activity is regulated posttranslationally.

## DISCUSSION

In response to prolonged or excessive immune activity, as seen in later stages of cancer, TNF-α produced by the tumor and/or its microenvironment is thought to promote many of the metabolic disorders associated with cachexia including altered glucose metabolism, lipid atrophy, and muscle wasting (*1*, *2*). While the metabolic functions of TNF-α were mainly studied in the context of disease, recent studies in flies reported a role of Egr/TNF and Grnd/TNFR in repressing systemic insulin signaling in response to nutritional stress (*21*), implying a more general role of TNF-TNFR signaling in regulating metabolism. Here, we provide the first example of a TNFR that is essential for restricting lipid catabolism in the adult gut in homeostatic conditions, by showing that knockdown of Wgn/TNFR in ECs results in autophagy-dependent mobilization of lipid stores in the gut and accelerated tissue turnover. The Wgn-dependent effect on lipolysis is mediated through the accumulation of cytoplasmic dTRAF3, suggesting that TRAFs are key regulators of metabolism. In line with this, ectopic expression of dTRAF3 was previously reported to trigger target of rapamycin (TOR)–dependent autophagy (*3*), while dTRAF2 associates with the autophagy protein Atg9, which is required for oxidative stress–induced tissue repair and autophagy in flies (*22*). The role of TNFRs and TRAFs in controlling energy homeostasis has not been addressed in mammals; however, on the basis of the high evolutionary conservation of the TNFR superfamily and TRAF proteins, it is likely that mammalian TNFRs also have beneficiary effects on energy homeostasis and gastrointestinal (GI) health.

How might Wgn control dTRAF3-mediated signaling? Our previous and current data show that Wgn is localized in intracellular vesicles in both developing (*12*) and adult tissues (Fig. 1, A and B, and fig. S1B), while dTRAF3 is restricted to the membrane in homeostatic conditions (Fig. 3I, and fig. S3, O to Q). Notably, upon forced expression, a fraction of Wgn localizes to the membrane, suggesting that Wgn cycles between the two compartments. Although Wgn is expressed at low levels in ECs, its depletion in these cells has marked effects on cytoplasmic dTRAF3 levels and dTRAF3-dependent processes, suggesting that Wgn might provide an enzymatic activity marking dTRAF3 for degradation. Notably, a previous study reported that knockdown of the E3 ubiquitin ligase, NOPO (TRIP in mammals), triggers the accumulation of cytoplasmic dTRAF3 and the activation of downstream effectors (fig. S3, U to W) (*3*). The authors found that NOPO interacts with dTRAF3 in a transient manner to promote its degradation and that cotransfection of dTRAF3 with an enzymatically inactive form of NOPO, but not wild-type NOPO, results in the accumulation of dTRAF3 in NOPO-positive vesicles (*3*). In mammals, NOPO/TRIP and TRAFs both interact with TNFRs, and therefore, it is plausible that Wgn facilitates NOPO-mediated degradation of internalized dTRAF3 to restrict lipid catabolism.

A recent study showed that Wgn localizes to late endosomes and lysosomes in the embryonic tracheal network, where it acts independently of Egr to promote degradation of the FGFR receptor, Breathless (Btl), and repress terminal cell differentiation (*11*). Intriguingly, the authors show that knockdown of Wgn results in the accumulation of Btl and its ligand, branchless (Bnl), in intracellular vesicles, suggesting that Wgn restricts Bnl/Btl signaling by preventing their accumulation in intracellular vesicles (*11*). The observation that Wgn suppresses signaling by restricting the accumulation of proteins in intracellular compartments in both the embryonic tracheal system and the adult gut opens up the exciting possibility that TNFRs could have a more general function in controlling protein localization and/or degradation and thereby regulate a broad spectrum of physiological processes independent of their canonical ligands. Deciphering how Wgn couple environmental cues with protein localization/degradation represents an exciting future area of research.

It was previously shown that bacterial-derived uracil induces the formation of dTRAF3^+^Cad99c^+^ vesicle signaling endosomes in a Hedgehog (Hh)– and dTRAF3-dependent manner triggering DUOX activity and ROS production (*3*, *23*, *24*). Assembly of TRAF3^+^Cad99c^+^ vesicles results in dTRAF3-mediated suppression of TOR activity to reduce lipogenesis, promote lipolysis, and support ROS production in this condition (*3*). Notably, EC-specific knockdown of Wgn did not trigger dTRAF3^+^Cad99c^+^ vesicle formation and ROS production, and hence, Wgn depletion does not recapitulate all the effects associated with infection-induced dTRAF3 activation (*3*). In line with this, Wgn depletion did not reduce the expression levels of the two lipogenesis enzymes, ACC1 and FAS (fig. S2, F and G), both targets of TOR, showing that TOR-dependent lipogenesis is not inhibited by Wgn. This suggests that Wgn knockdown triggers dTRAF3-dependent lipolysis independently of dTRAF3’s role in suppressing TOR-mediated lipogenesis in dTRAF3^+^Cad99c^+^ vesicles. Although Wgn knockdown is not sufficient to trigger the formation of dTRAF3^+^Cad99C^+^ endosomes and ROS production in homeostatic conditions, its inactivation might still be required downstream or in parallel with infection-induced Hh-mediated Cad99c expression and dTRAF3^+^Cad99c^+^ vesicle formation. Alternatively, the formation of dTRAF3^+^Cad99C^+^ endosomes protects dTRAF3 from Wgn-dependent degradation in this condition (*23*). The role of dTRAF3 in rewiring lipid metabolism toward catabolism is essential for coping with infections, as dTRAF3 depleted flies not only display reduced infection-induced lipolysis (Fig. 5N) but also are highly susceptible to oral infection (*3*).

Previous studies in flies have suggested that innate gut immunity is not controlled by TNF signaling (*6*) but rather depends on PGN/PGRP-dependent activation of the IMD–dTAK1–Relish/NF-κB pathway. Nevertheless, the fly IMD and mammalian TNF-α/TNFR1 pathways are notably similar (fig. S1A). Here, we show that knockdown of Wgn is sufficient to activate NF-κB signaling in the gut (Fig. 4, A and B, and fig. S4, A and B), demonstrating that the role of TNFRs in regulating Relish/NF-κB–mediated immune processes is conserved between flies and mammals. Intriguingly, *egr* mutant flies display elevated levels of AMPs, which is recapitulated by knockdown of Egr in progenitor cells, but not ECs (Fig. 4, C and D, and fig. S4, C and D). This shows that Egr derived from progenitor cells, and possibly other non-intestinal sources, signals non-autonomously through Wgn in ECs to restrict immunity. Wgn suppresses immunity through an inhibitory effect on the IMD pathway member, dTAK1, and this is mediated by its canonical downstream effector dTRAF2 (TRAF6 in mammals). Hence, dTRAF2 depletion phenocopies the induction of AMPs caused by knockdown of Wgn and can be rescued by dTAK1 knockdown (Fig. 4, K and L). We further show that both Bsk/JNK and Relish/NF-κB are required for the AMP induction associated with Wgn loss of function in a nonredundant manner (Fig. 4, G to J). Therefore, contrary to the current view, our data demonstrate a conserved role of TNFR signaling in regulating innate immunity, although the mechanism underpinning TNFR-mediated immune regulations differ between flies and mammals. Thus, while mammalian TRAFs are believed to couple the ligand-dependent activation of surface receptors, such as TNFRs, with JNK/MAPK and NF-κB signaling to trigger inflammation and immunity, Egr/TNF and Wgn/TNFR serve to restrict Relish/NF-κB signaling and immunity in the adult fly gut. Intriguingly, the ability of Wgn to suppress JNK-mediated immunity contrasts with the well-defined role of Egr in promoting JNK-dependent apoptosis and tumorigenesis in the developing eye compound (*6*, *25*). Although early studies suggested that Egr signals through Wgn to promote JNK-dependent apoptosis (*26*), subsequent studies demonstrated that Grnd/TNFR, and not Wgn, is required downstream of Egr to activate JNK signaling in this tissue (*5*). How binding of Egr to different TNFRs translates into opposing effects on JNK-mediated processes warrants further investigation. The induction of AMPs triggered by dTRAF2 loss of function does not depend on IMD (Fig. 4, M and N), suggesting that dTAK1 activity and immunity are not controlled by PGN/PGRP signaling in homeostatic conditions. Hence, while mammalian TNFR1 uses both RIP1 (homologous to IMD) and TRAF2 in the regulation of TAK1–NF-κB–dependent processes, our data suggest that IMD and dTRAF2 represent separate upstream branches that converge on dTAK1 to regulate Rel/NF-κB–mediated immunity. Although the PGN/PGRP-IMD branch is likely to be the main inducer of immunity in response to infection, the observation that overexpression of dTRAF2 is sufficient to suppress infection-induced immunity (Fig. 5O) suggests that inactivation of the Wgn-dTRAF2 pathway is required for full activation of the NF-κB pathway in this condition. It remains to be determined whether suppression of Wgn-dTRAF2–dependent signaling is mediated through an inhibitory effect on the ligand, Egr (e.g., its secretion), Wgn, and/or dTRAF2.

Although EC-specific knockdown of Wgn activates dTRAF3 and dTAK1-Bsk/JNK signaling to promote *upd3* expression and proliferation in homeostatic conditions, dTRAF3 is not required for the proliferative response associated with oral infections (fig. S5K). We found that like Egr, Wgn is required in progenitor cells to promote regenerative growth (Fig. 5P). Hence, while Wgn is required in a ligand-independent manner to maintain tissue homeostasis in ECs, it likely acts as a receptor for Egr in progenitor cells to promote ISC divisions during tissue repair.

As anti-TNF therapies are now widely used to treat chronic inflammatory diseases, such as IBD, there is an urgency to better understand the homeostatic functions of TNF-TNFR signaling and how these might be affected by anti-TNF drug regimes. This is emphasized by case reports from the rheumatology fields showing that anti-TNF drugs can cause paradoxical adverse effects on the GI tract resembling some aspects of IBD, suggesting that TNF signaling also promotes GI health [reviewed in (*27*)]. Here, we show that the fly TNFR, Wgn, is required in the gut epithelium to maintain immunometabolism homeostasis. The notable observation that Wgn/TNFR has important metabolic and anti-inflammatory functions in homeostatic conditions opens the possibility that mammalian members of the TNFR superfamily might also carry out protective, and possibly ligand-independent, functions in the GI tract of healthy individuals. Therefore, future studies should aim at characterizing the putative beneficiary roles of mammalian TNFRs and TRAFs in controlling metabolism, immunity, and tissue repair in homeostatic conditions.

## MATERIALS AND METHODS

### Drosophila stocks and husbandry

Flies were maintained on a standard cornmeal medium [containing cornmeal (82 g/liter), sucrose (60 g/liter), yeast (34 g/liter), agar (8 g/liter), propionic acid (4.8 ml/liter), and methyl-4-hydroxybenzoate (1.6 g/liter)] at 25°C and 60% relative humidity under normal photoperiod (12-hour light:12-hour dark cycles). Genotypes carrying temperature-sensitive GAL80 (GAL80^ts^) were raised through eclosion at 18°C. Virgin flies were collected and kept at 18°C for 4 to 7 days then transferred to 29°C for 10 to 14 days to induce upstream activating sequence (UAS) expression before the start of experiments. Flies were flipped onto fresh medium every second day.

### Generation of wgn^attP^ and wgn^CRISPR^

To generate a *wgn* null mutant, an approach combining the CRISPR technique and homologous recombination was used as described in (*28*). Double-strand breaks were induced by the CRISPR technique using single-stranded guide (sg)RNAs and a Cas9-encoding plasmid. For optimal targeting of the *wgn* locus, sgRNA target sequences were selected as 20-nt sequences preceding an NGG PAM sequence in the genome (GN20GG). To generate pCFD4{wgn^attP^}, gRNAs targeting sequences immediately before and after exon I in the *wgn* locus were cloned into the tandem gRNA expression vector, pCFD4 [gift from S. Bullock, MRC Laboratory of Molecular Biology, Cambridge, UK (Addgene plasmid #49411)], using the *wgn*-5′-pCFD4-FORWARD: 5′- TAT ATA GGA AAG ATA TCC GGG TGA ACT TCg aat tag cat gcc tat gac cgt GTT TTA GAG CTA GAA ATA GCA AG-3′ and *wgn*-3′-pCFD4-REVERSE: 5′- ATT TTA ACT TGC TAT TTC TAG CTC TAA AAC tac gtc gcc act ggc gca tgc GAC GTT AAA TTG AAA ATA GGT C-3′ (*wgn*-specific sequences are in lower case) primers as described in www.crisprflydesign.org/wp-content/uploads/2014/06/Cloning-with-pCFD4.pdf. For homologous recombination, two homology arms were amplified from genomic DNA using the following primers: for homology arm I, sense: 5′- CAC C**GC GGC CGC** cat ggc gga ggc ccc-3′ and antisense: 5′- **GGT ACC** ggc atg cta att ctg ttg ttg ata atgc -3′, for homology arm II, sense: 5′- CAC C**AC TAG TG**T ATG GAG CGT CGC CTG GAC -3′ and antisense: 5′- **GGC GCG CC**g ggc ggg gcg gga g -3′ (in bold are the added restrictions sites used for cloning into pTV2). The resulting polymerase chain reaction (PCR) products were digested and cloned into the pTV2 vector (gift from C. Alexandre and J.-P. Vincent, the Francis Crick Institute, London, UK) to generate pTV2{wgn, mini-white}. To facilitate homologous recombination, embryos were injected with pTV2{wgn, mini-white} in the presence of pCFD4{wgn^attP^} and a Cas9-containing plasmid. After confirmed targeting, the resulting strain harbors a deletion of the entire first and second coding exons and an *attP* integration site in the *wgn* locus and is referred to as w*gn^attP^* in the manuscript. To generate wgn^CRISPR^, gRNAs targeting the same sequences than the ones used for *wgn^attP^* mutant were cloned into pCFD6 (Addgene plasmid #73915), using the *wgn*-5′-pCFD6-FORWARD: 5′- CGG CCC GGG TTC GAT TCC CGG CCG ATG CAa tta gca tgc cta tga ccg tGT TTC AGA GCT ATG CTG GAA AC-3′ and *wgn*-3′-pCFD6-REVERSE: 5′- ATT TTA ACT TGC TAT TTC TAG CTC TAA AAC tac gtc gcc act ggc gca tgT GCA CCA GCC GGG AAT CGA ACC-3′ (*wgn*-specific sequences are in lower case). pCFD6-wgn has been coinjected with a PhiC31 integrase helper vector and is referred to as w*gn^CRISPR^* in the manuscript.

### Transgenic flies

To generate the *UAS-dTRAF3* and UAS*-Wgn* lines, *dTRAF3* coding sequences were PCR-amplified from BDGP EST complementary DNA clones RE66324 and cloned into the pENTR/D-TOPO vector using the following gene-specific sense primer CACC ATG CGA TCC CAT CTG AAG GAG TG and antisense primer TTA TTT TAG AAC TCG AAC CTC GAT GAA CAT G. *wgn* coding sequences were PCR-amplified and cloned into the pENTR/D-TOPO vector using the following gene-specific sense primer CAC CAT GAT GCC GCC AAG ACT GCC and antisense primer TCA GCC CTT CAG GCC GG. *dtraf3* and *wgn* coding sequences were subcloned into pUASg.attB and *pUASattB-dTRAF3*, and *pUASattB-wgn* constructs were introduced into the germ line by injections in the presence of the PhiC31 integrase and inserted in the 86F8-landing site on the 3R chromosome (Bloomington *Drosophila* Stock Center, BL24749, BestGene). *UAS-dTRAF3-V5*, *UAS-dTRAF3^RNAi^*, *dTRAF3-V5 genomic construct*, and *dTRAF3* mutant flies were a gift from W.-J. Lee (School of Biological Sciences, Seoul National University, South Korea). UAS-dTRAF1 flies were a gift from M. Leptin (Institute for Genetics, University of Cologne, Cologne, Germany). *wgn-lacZ.E1197* flies were a gift from M. Frasch (Department of Biology, University of Erlangen-Nuremberg, Erlanger, Germany), and *w; myo1A-Gal4; tubGal80ts UAS-GFP/TM6b* stock was a gift from N. Tapon (Crick Institute, London, UK). *upd3-lacZ* stock was a gift of B. Lemaitre [École Polytechnique Fédérale de Lausanne (EPFL), Lausanne, Switzerland]. *mex-Gal4* was a gift of B. Charroux [Marseille Developmental Biology Institute (IBDM), Marseille, France]. *egr^3AG^* was a gift from L. Johnston (Department of Genetics and Development, NY, USA). Following stocks are from the Vienna Drosophila RNAi Center (UAS-wgn^RNAi^ v9152, *wgn-GFP* v318644, *UAS-atg1^RNAi^* v16133, *UAS-dTAK1^RNAi^* v101357, *UAS-rel^RNAi^* v108469, *UAS-dTRAF1^RNAi^* v110776, *UAS-dTRAF2^RNAi^* v16125, *UAS-NOPO^RNAi^* v104477, *UAS-egr^RNAi^* v108814, *UAS-bsk^RNAi^* v104569, and *UAS-imd* v9253). The following stocks are from the Bloomington Stock Center [*mef2-Gal4* BL25756, *esg-Gal4, UAS-GFP BL67085, tub-Gal80^ts^* BL7108 and BL7017, *UAS-Cas9.P2* BL58985, *yw hsFLP* BL7*, yw act-Gal4(FRT.CD2); UAS-GFP* BL39760, *w; act-Gal4(FRT.CD2), UAS-RFP* BL30558, *UAS-dTRAF2* BL58991, *esg-lacZ* BL10359, *wgn^Df^* BL8036, *UAS-dTAK1^RNAi^* BL33404]. *grnd^KO^* and *wgn^KO^* have been previously published (*5*).

### Nile red stainings

Guts were dissected in phosphate-buffered saline (PBS) and then fixed for 30 to 45 min with 4% paraformaldehyde under gentle agitation. Fixed guts were rinsed two times with PBS, then incubated in the dark with Nile red solution [diluted 1:300 in 1× PBS from a stock solution (2.5 μg/ml)] for 30 to 45 min at room temperature with slow shaking (maximum of 50 rpm), and washed once in PBS. Guts were then carefully mounted on glass slides using Fluoroshield (Sigma-Aldrich, F6182) or Vectashield (H-1200, Vector laboratories) mounting media containing 4′,6-diamidino-2-phenylindole. Mounted guts were immediately scanned using a confocal microscope. The same protocol was followed for Nile red stainings of fat bodies. Tissues were imaged on a Zeiss LSM-900 confocal microscope using a 20× objective in the Zen software package. Postimaging analysis was performed using the open-source Fiji software package. Between 10 and 15 guts per genotype were used in each experiment. Images were taken in the R2 or in the R4bc regions as defined in figure legends. The compartmentalization of the gut into five major regions (R1 to R5) and 14 subregions was described in (*29*). The R2 and R4 regions express high levels of genes associated with lipid metabolism compared with R1, R3, and R5 (*29*).

### LipidTox stainings

The same protocol of Nile red was performed for LipidTox staining. HCS LipidTOX Deep Red neutral lipid stain (Invitrogen, H34477) was used in all experiments. Guts were incubated with LipidTox solution (1/300 in 1× PBS) for 30 to 45 min at room temperature with gentle shaking (maximum of 50 rpm). LipidTOX Deep Red neutral lipid stain can be imaged with filter sets appropriate. LipidTOX Deep Red neutral lipid stain was imaged with filter sets appropriate for Alexa Fluor 647 dye. Between 10 and 15 guts per genotype were used in each experiment. Images were taken in the R4bc region as defined in figure legends.

### LysoTracker staining

Guts were rapidly dissected in PBS and then fixed for 5 min with 4% paraformaldehyde without agitation. Guts were rinsed with PBS and then incubated for 5 min with LysoTracker solution (Invitrogen, L12492) diluted in PBS (1/1000). All the steps were performed in the dark. Guts were mounted in Fluoroshield mounting medium and imaged immediately on a Zeiss LSM-900 confocal microscope using a 40× objective. Between 10 and 15 guts per genotype were used in each experiment. Images were taken in the R4bc region.

### Quantification analyses

The quantification of lipid droplets and lysosome was conducted using Fiji software as described in (*30*). A region of interest was manually selected for each image in the specified regions of the gut, and the area was determined. Lipid droplets or lysosomes were identified by maximum entropy thresholding and converted to binary images to determine particle sizes (in square micrometers) and numbers using the “Analyze Particles” plug-in. The density of the lipid droplets or lysosomes numbers was calculated as the number of droplets per unit of area.

### Immunostainings

Guts were dissected in PBS and then fixed for 30 to 45 min with 4% paraformaldehyde with gentle agitation. Fixed guts were washed one time in PBS and then washed 3 × 10 min in PBS containing 0.1% Triton (PBS-T). Guts were then blocked for 2 hours in PBS-T containing 10% fetal calf serum (FCS). Incubations with primary antibodies were done overnight in blocking at 4°C. The next day, guts were washed with PBS-T 2 × 10 min and blocked in PBS-T containing 10% FCS for 20 min. Secondary staining was performed at room temperature for 2 to 4 hours. The primary antibodies and dilutions used were: 1:250 for mouse anti-Wgn (*5*), 1:250 for guinea pig anti-Grnd (*5*), 1:10,000 for chicken anti-GFP (Abcam, 13970), 1:300 for rabbit anti-dTRAF3 (this paper), 1:1000 for rabbit anti-PH3 (Milipore, 06-570), 1:500 for mouse anti–β-Gal [Developmental Studies Hybridoma Bank (DSHB), 40-1A], 1:300 for mouse anti-V5 (Invitrogen, R96025), and 1:100 for mouse anti-Armadillo (DSHB, N2-7A1) and for mouse anti-Discs Large (DSHB, 4F3). For the secondary fluorophore-conjugated antibodies, the dilutions used were as follows: 1:1000 for Alexa Fluor Plus 488–conjugated goat anti-mouse (Thermo Fisher Scientific, #A32723), 1:1000 for Alexa Fluor 488–conjugated goat anti-rabbit (Thermo Fisher Scientific, #A11008), 1:1000 for Alexa Fluor 555–conjugated goat anti-mouse (Thermo Fisher Scientific, #A32727), 1:1000 for Alexa Fluor 555–conjugated goat anti-rabbit (Thermo Fisher Scientific, #A21428), and 1:500 and 1:1000 for Alexa Fluor 647–conjugated goat anti-rabbit (Thermo Fisher Scientific, #A32733). Tissues were imaged on a Zeiss LSM-900 confocal microscope using a 20× or 40× objectives in the Zen software package. Postimaging analysis was performed using the open-source Fiji software package. Between 10 and 20 guts per genotype were used in each single experiment, and representative images for each experiment were chosen. Images were processed in either Adobe Photoshop or Fiji software. Images were taken R4bc region.

### ROS measurements

To measure ROS levels, flies were orally administered a 5% sucrose solution containing a hypochlorous acid (HOCL)-specific rhodamine-based R19S dye (50 mM) for 30 min. Next, midguts were dissected and fixed in 4% paraformaldehyde for 1 hour. HOCL production was visualized using a Zeiss LSM-900 confocal microscope. The number of R19S-positive guts per total number of intestinal samples is shown as percentage of ROS-positive guts. An intestine was considered as positive for R19S labeling when at least 10% of the anterior midgut displayed a fluorescent labeling of the EC membrane.

### Flip-out clones

For flip-out clones, *yw, hs-flp; UAS-X-RNAi males* were crossed with *w; act>CD2>GAL4; UAS-GFP* or *w; act>CD2>GAL4, UAS-RFP* females. Female virgin progeny was sorted, kept 4 to 7 days at 18°C, and then heat-shocked for 10 to 30 min at 37°C, before being transferred to 29°C for 7 to 10 days before dissection. Between 5 to 15 guts per genotype containing individual and nonconfluent clones were used in each experiment (wgn RNAi: 48 clones per gut in average; atg1 + wgn RNAi: 33 clones per gut in average; wgn CRISPR clones: 15 clones per gut in average). Images were taken in the R4bc.

### TRAF3 antibody

To generate purified antisera specific for the dTRAF3 protein, two peptides, TDVGNIRKQNQVVED and TKLGNSDYVTSKQAT, corresponding to amino acids 140 to 154 and 161 to 176, respectively, were used as immunogens in Rabbit (Eurogentec).

### Starvation-survival assays

Virgin female flies were sorted 10 by 10 and kept for 7 days at 18°C before being transferred to 29°C for 7 days. Flies were then transferred to starvation medium (1% agar in water) and kept at 29°C. Flies were flipped onto fresh starvation food every day. The number of dead animals was assessed each 6 hours until the completion of the assay. For each genotype, 50 to 100 flies were used. Statistical significance was calculated using the Kaplan-Meier log-rank survival functions of the Prism program (GraphPad).

### Longevity assays

Newly emerged flies were collected and kept 48 hours at 25°C to mate. On the third day, female flies were sorted 10 by 10 into vials containing metabolic food containing sucrose (90 g/liter), yeast (80 g/liter), agar (10 g/liter), 0.5% propionic acid, and 0.15% methyl-4-hydroxybenzoate at 25°C and 60% relative humidity under normal photoperiod (12-hour light:12-hour dark) and transferred to 29°C. Flies were flipped onto fresh food every second day. The number of dead animals was assessed each 2 days until the completion of the assay. For each genotype, 50 to 120 flies were used. The *wgn* RNAi stock was backcrossed six times before performing the survival assays. Statistical significance was calculated using the Kaplan-Meier log-rank survival functions of the Prism program (GraphPad).

### Metabolite measurements

The protocol followed is described in (*31*). For whole-body and fat body TAG measurements, four adult flies with or five without guts and ovaries, respectively, were homogenized in PBS buffer with 0.05% Tween-20 (Sigma-Aldrich, #1379) using a TissueLyser LT (Qiagen) bead mill with 5-mm stainless-steel beads. An aliquot of the homogenate was used for bicinchoninic-acid protein-level determination using commercial components (Sigma-Aldrich, #B9643, #C2284, and #P0914). Tri- and diacylglycerides were measured by cleaving their ester bonds using triglyceride reagent (Sigma-Aldrich, #T2449) to liberate glycerol, which was measured using Free Glycerol Reagent (Sigma-Aldrich, #F6428) in a colorimetric assay. Absorbance was read at 540 nm using an EnSight multimode plate reader (PerkinElmer). TAG was quantified using corresponding glycerol standard curves. All measurements were normalized to the number of animals used for each sample.

### Feeding assays

Short-term consumption was measured using a spectrophotometric dye-feeding assay (*32*, *33*). At the time of the main morning meal (1 hour after lights-on in the incubator, ZT0), flies were transferred without anesthesia to nutrient-balanced sugar-yeast food (*34*) [sucrose (90 g/liter), yeast (80 g/liter), and agar (10 g/liter), with propionic acid (1 ml/liter) and methyl-4-hydroxybenzoate (1 g/liter) containing 0.5% erioglaucine (brilliant blue R, FD&C Blue No. 1, Sigma-Aldrich, #861146)] and allowed to feed for 2 hours. Other flies were allowed to feed on undyed medium for use in baselining spectrophotometric measurements. Fifteen sets of three animals for each genotype were homogenized in 50 μl of 50 mM PB (pH 7.5) using a TissueLyser LT (Qiagen) bead mill with 5-mm stainless-steel beads (Qiagen, #69989). Insoluble debris was pelleted by centrifugation, 50 μl of the supernatant was transferred to a 384-well plate well, and absorbance at 629 nm was measured using an Ensight multi-mode plate reader (PerkinElmer). Readings were calibrated against an erioglaucine standard curve to compute the amount of food consumed per fly over 2 hours.

Long-term consumption was monitored using the CApillary FEeder assay (CAFE assay) (*35*). Each fly was placed into a 2-ml Eppendorf tube with a 5-μl microcapillary (Hirschmann) inserted through a hole in the lid; the capillary was filled with a liquid sugar-yeast medium [sucrose (50 g/liter) and yeast extract (50 g/liter), with propionic acid (1 ml/liter) and methyl-4-hydroxybenzoate (1 g/liter)]. To minimize evaporation, capillary-equipped tubes were kept in a moist chamber, and fly-free tubes were used to control for the level of evaporation. The amount of food consumed was determined by measuring the food level within the capillary tube every 24 hours for 4 days. Tubes containing no flies were used as controls for evaporation. Twenty flies were individually scored for each genotype.

### Oral *Ecc15* and P.e. infections

For oral infection, 7-day-old *w^1118^* virgin females were starved for 2 hours and then flipped onto fly medium covered with filter disks soaked in a 1:1 mix of bacterial pellets and 5% sucrose (concentration, 1.10^8^ colony-forming units per fly) or 5% sucrose solution alone (control). For PH3 measurements, flies were infected with *Ecc15* for 16 hours. For quantification of infection-induced lipid depletion (Fig. 5N and fig. S5O), flies were infected every 3 days with *Ecc15* over the course of 1 week. For quantitative PCR analyses, flies were infected with Ecc15 for 4 hours (fig. S5N) or 16 hours (Fig. 5, O and P, and fig. S5P) or P.e. for 16 hours (fig. S5L).

### Quantitative RT-PCR

Dissected adult guts were transferred directly into lysis buffer and frozen in liquid nitrogen. Total RNA was extracted from dissected tissues using a RNeasy microkit (Qiagen) according to the manufacturer’s protocol. RNA samples (1 μg per reaction) were treated with deoxyribonuclease and reverse-transcribed using SuperScript II reverse transcriptase (Invitrogen), and the generated cDNAs were used for real-time reverse transcription (RT)–PCR (StepOne Plus; Applied Biosystems) using RealQ Plus 2x Master Mix Green (Ampliqon), with 8 ng of cDNA template and a primer concentration of 500 nM. Samples were normalized to levels of ribosomal protein (rp)49 transcript levels. Four to six separate biological samples were collected for each experiment, and triplicate measurements were performed. The following primers were used:

*Pepck_F* 5′- TCA ATG GCG AAT CCT GCT AC-3′, *Pepck_R* 5′- TCC TTC ACG TCC ACC TTA TCC-3′; *Fbp_F* 5′- AGC AAT TGT CCT GGG TCT GG-3′, *Fbp_R* 5′- CCA ATC CGC TGC ATA TCC CT-3′; *Tsp1_F* 5′- CGT GTG ACA TCG TCG GAT ATT-3′, *Tsp1_R* 5′- AGT GTC GTT CCA CCC ATT TC-3′; *Wgn2_F* 5′-CCG AAA GTC CTC CGA GTA TG-3′, *Wgn2_R* 5′-TAA AGA ACG CCA GTC TGC C-3′; *Dipt_F* 5′- GCT GCG CAA TCG CTT CTA CT -3′, *Dipt_R* 5′- TGG TGG AGT GGG CTT CAT G -3′; *Cec2A_F* 5′- GGA CAA TCG GAA GCT GGT T − 3′, *Cec2A_R* 5′- TGT GCT GAC CAA CAC GTT C -3′; *Upd3_F* 5′-ACC ACC AAT GCG GAC AAG -3′, *Upd3_R* 5′-ATT CAG ACG GGG CAG GAA-3′; *Puc_F* 5′-GCC ACA TCA GAA CAT CAA GC-3′, *Puc_R* 5′-CCG TTT TCC GTG CAT CTT-3′; *dtraf3_F* 5′- TTG CCA GTG TCC AGA ATA TAA G -3′, *dtraf3_R* 5′- ATC CAG CCG TAG AGC ATA G -3′; *Lip3_F* 5′- ATC AAG TCC GCC CAT CTT CT -3′, *Lip3_R* 5′- CTC TAT GCC CAA ATC CTG CT-3′; *Lip4_F* 5′- ACA AGG GAC TTG GTT ACC TG-3′, *Lip4_R* 5′- TGG GTG CTA TAC TTA ACA TGC T-3′; *Brummer_F* 5′- ACG CAC AGC AGC GAC ATG TAT-3′, *Brummer_R* 5′- CTT TTC GCT TTG CTA CGA GCC-3′; *Margo_F* 5′- ACA CCG AAC TGA TTC CGA AC-3′, *Margo_R* 5′- ATC CAC CAT TGG CAA ACA T-3′; *Fas_F* 5′- CGT ACG ACC CCT CTG TTG AT AC-3′, *Fas_R* 5′- AGT GCA AGT TAC CGG GAA T-3′; *Acc_F* 5′- TAA CAA CGG AGT CAC CCA CA -3′, *Acc_R* 5′- CAG GTC ACA ACC GAT GTA CG-3′; *Atg1_F* 5′- GAG TAT TGC AAT GGC GGC GAC T-3′, *Atg1_R* 5′- CAG GAA TCG CGC AAA CCC AA-3′; *Atg8a_F* 5′- GCA AAT ATC CAG ACC GTG TGC C-3′, *Atg8a_R* 5′- AGC CCA TGG TAG CCG ATG-3′; *Atg4b_F* 5′- CAT CCA CTT GGA TCC GCA CT-3′, *Atg4b_R* 5′- GTG AAA CGA GTG CAG GGA GA-3′; *Lsd1_F* 5′- ATG TTT GGC CAC AAA AGC CC-3′, *Lsd1_R* 5′- GGG AGA AGC GTT GAC CAT GA-3′; *SH3PX1_F* 5′- TTC CCC TCT CGG ATC GAC TT-3′, *SH3PX1_R* 5′- CCC TTG TGC GTG GAG AAG AT-3.

### Statistics

Statistics were performed using the Prism software (GraphPad). All data were assessed for normality using Shapiro-Wilkchecks before running statistical tests. Multiple comparisons were analyzed using analysis of variance (ANOVA), and pairwise comparisons were made using two-tailed *t* tests for normally distributed data or two-tailed Mann-Whitney *U* tests for other data. Starvation-survival curves and longevity curves were analyzed using Kaplan-Meier log rank tests. Significance is shown in figures as not significant (ns), *P* > 0.05; *P ≤ 0.05; **P ≤ 0.01; ***P ≤ 0.001.

### Genotypes

Figure 1:

A: *w; mex-Gal4/+; tubGal80*^*ts*^*/ UAS-wgn*

B: *w; +; +*

C: *yw, wgn*^*K0*^*/+*; *+ ; +*

D: *w, wgn*^*attP*^*/+*; *+ ; +*

E: yw, *wgn*^*K0*^*/w, wgn*^*attP*^;* + ; +*

G: *w; mex-Gal4/+; tubGal80*^*ts*^*/+*

H: w; +; *UAS-wgn*^*RNAi*^/+

I: *w; mex-Gal4/+; tubGal80*^*ts*^* / UAS-wgn*^*RNAi*^

K to M: *yw act-Gal4(FRT.CD2)/ yw hsFLP; UAS-GFP/+; UAS-wgn*^*RNAi*^/+

O to Q: *yw act-Gal4(FRT.CD2)/ yw hsFLP; UAS-GFP/ UAS-Cas9P2; pCFD6*^*wgn*^/+

Figure 2: 

D: w; +; *UAS-wgn*^*RNAi*^/+

E: *w; mex-Gal4/+; tubGal80*^*ts*^* / +*

F: *w; mex-Gal4/+; tubGal80*^*ts*^* / UAS-wgn*^*RNAi*^

G to I: *yw act-Gal4(FRT.CD2)/ yw hsFLP; UAS-GFP/ UAS-atg1*^*RNAi*^*; UAS-wgn*^*RNAi*^/+

Figure 3: 

A: *w; mex-Gal4/+; tubGal80*^*ts*^*/+*

B: w; +; *UAS-dTRAF3-V5*/+

C: *w; mex-Gal4/+; tubGal80*^*ts*^* / UAS-dTRAF3-V5*

F to H: * yw hsFLP/ w; +; UAS-wgn*^*RNAi*^/ *act-Gal4(FRT.CD2), UAS-RFP*

I: *w; mex-Gal4/+; tubGal80*^*ts*^*/+*

J: *w; mex-Gal4/+; tubGal80*^*ts*^* / UAS-wgn*^*RNAi*^

K: *w; mex-Gal4/+; tubGal80*^*ts*^*/+*

L: *w; mex-Gal4/+; tubGal80*^*ts*^* / UAS-wgn*^*RNAi*^

M: *w; mex-Gal4/ +; tubGal80*^*ts*^* / UAS-wgn*^*RNAi*^*, UAS-dTRAF3*^*RNAi*^

N: *w; +; UAS-wgn*^*RNAi*^*, UAS-dTRAF3*^*RNAi*^*/ +*

O: *w; mex-Gal4/ UAS-atg1*^*RNAi*^*; UAS-wgn*^*RNAi*^*/ tubGal80*^*ts*^

P*: w; UAS-atg1*^*RNAi*^* / +; UAS-wgn*^*RNAi*^* / +*

R: *w; mex-Gal4/+; tubGal80*^*ts*^*/+*

S: *w; mex-Gal4/+; tubGal80*^*ts*^* / UAS-wgn*^*RNAi*^

T: *w; mex-Gal4/ +; tubGal80*^*ts*^* / UAS-wgn*^*RNAi*^*, UAS-dTRAF3*^*RNAi*^

U: *w; mex-Gal4/ +; tubGal80*^*ts*^* / UAS-dTRAF3*^*RNAi*^

V: *w; +; UAS-wgn*^*RNAi*^*, UAS-dTRAF3*^*RNAi*^*/ +*

Figure 5: 

A: *w; mex-Gal4/ esg-LacZ; tubGal80*^*ts*^*/+*

B: *w; mex-Gal4/ esg-LacZ; tubGal80*^*ts*^* / UAS-wgn*^*RNAi*^

D: *w; mex-Gal4/ upd3-LacZ; tubGal80*^*ts*^*/+*

E: *w; mex-Gal4/ upd3-LacZ; tubGal80*^*ts*^* / UAS-wgn*^*RNAi*^

Figure S1: 

B: *+; + *;* wgn-GFP / +*

C*: +;* wgn-lacZ.E1197 /+; +

D: *w; tubGal80*^*ts*^*/+; mef2-Gal4/+;*

E: *w; tubGal80*^*ts*^*/+; mef2-Gal4/ UAS-wgn*^*RNAi*^

H:* w; grnd*^*K0*^*/+*;* + *

I:* w; grnd*^*K0*^*/grnd*^*K0*^;* + *

K:* w; egr*^*3*^*/+*;* + *

L:* w; egr*^*3*^*/egr*^*3*^;* + *

N: *w; mex-Gal4/+; tubGal80*^*ts*^*/+*

O: w; +; *UAS-wgn*^*RNAi*^/+

P: *w; mex-Gal4/+; tubGal80*^*ts*^* / UAS-wgn*^*RNAi*^

Q: *w; tubGal80*^*ts*^*/+; mef2-Gal4/+;*

R: *w; tubGal80*^*ts*^*/+; mef2-Gal4/ UAS-wgn*^*RNAi*^

Figure S3: 

A: *w; mex-Gal4/+; tubGal80*^*ts*^*/+*

B: w; *UAS-dTRAF1*^*RNAi*^/+; +

C: *w; mex-Gal4/ UAS-dTRAF1*^*RNAi*^*; tubGal80*^*ts*^*/+*

D: w; *UAS-dTRAF2*^*RNAi*^/+; +

E: *w; mex-Gal4/ UAS-dTRAF2*^*RNAi*^*; tubGal80*^*ts*^*/+*

G: *w; mex-Gal4/+; tubGal80*^*ts*^*/+*

H: w; +; *UAS-dTRAF1*/+

I: *w; mex-Gal4/+ ; tubGal80*^*ts*^*/ UAS-dTRAF1*

K: *w; mex-Gal4/+; tubGal80*^*ts*^*/+*

L: w; *UAS-dTRAF2*/+; +

M: *w; mex-Gal4/ UAS-dTRAF2; tubGal80*^*ts*^*/+*

O to Q*: dTRAF3-V5* (genomic construct) 

R*: dTRAF3*^*-/-*^*; +; +*

U to W: *yw act-Gal4(FRT.CD2)/ yw hsFLP; UAS-GFP/ NOPO*^*RNA*^*i; +*

Figure S4: 

N to P: *yw act-Gal4(FRT.CD2)/ yw hsFLP; UAS-GFP/+; UAS-wgn*^*RNAi*^/+

Q to S: *yw act-Gal4(FRT.CD2)/ yw hsFLP; UAS-GFP/ UAS-dTak1*^*RNAi*^*; UAS-wgn*^*RNAi*^/+

## Notes

View/request a protocol for this paper from *Bio-protocol*.
