# Supplementary material for: The Drosophila tumor necrosis factor receptor, Wengen, couples energy expenditure with gut immunity
Source: Sci Adv. 2023 Jun 9;9(23):eadd4977. doi: 10.1126/sciadv.add4977 (PMC13155476; doi:10.1126/sciadv.add4977)
Supplement: Supplementary file 1 — Figs. S1 to S5 [file sciadv.add4977_sm.pdf]

Science Advances
AAAS

Supplementary Materials for

The Drosophila Tumor Necrosis Factor Receptor, Wengen, couples energy expenditure with gut immunity

Rihab Loudhaief *et al.*

Corresponding author: Julien Colombani, julien.colombani@bio.ku.dk;
Ditte S. Andersen, ditte.andersen@bio.ku.dk

Sci. Adv. **9**, eadd4977 (2023)
DOI: 10.1126/sciadv.add4977

This PDF file includes:

Figs. S1 to S5

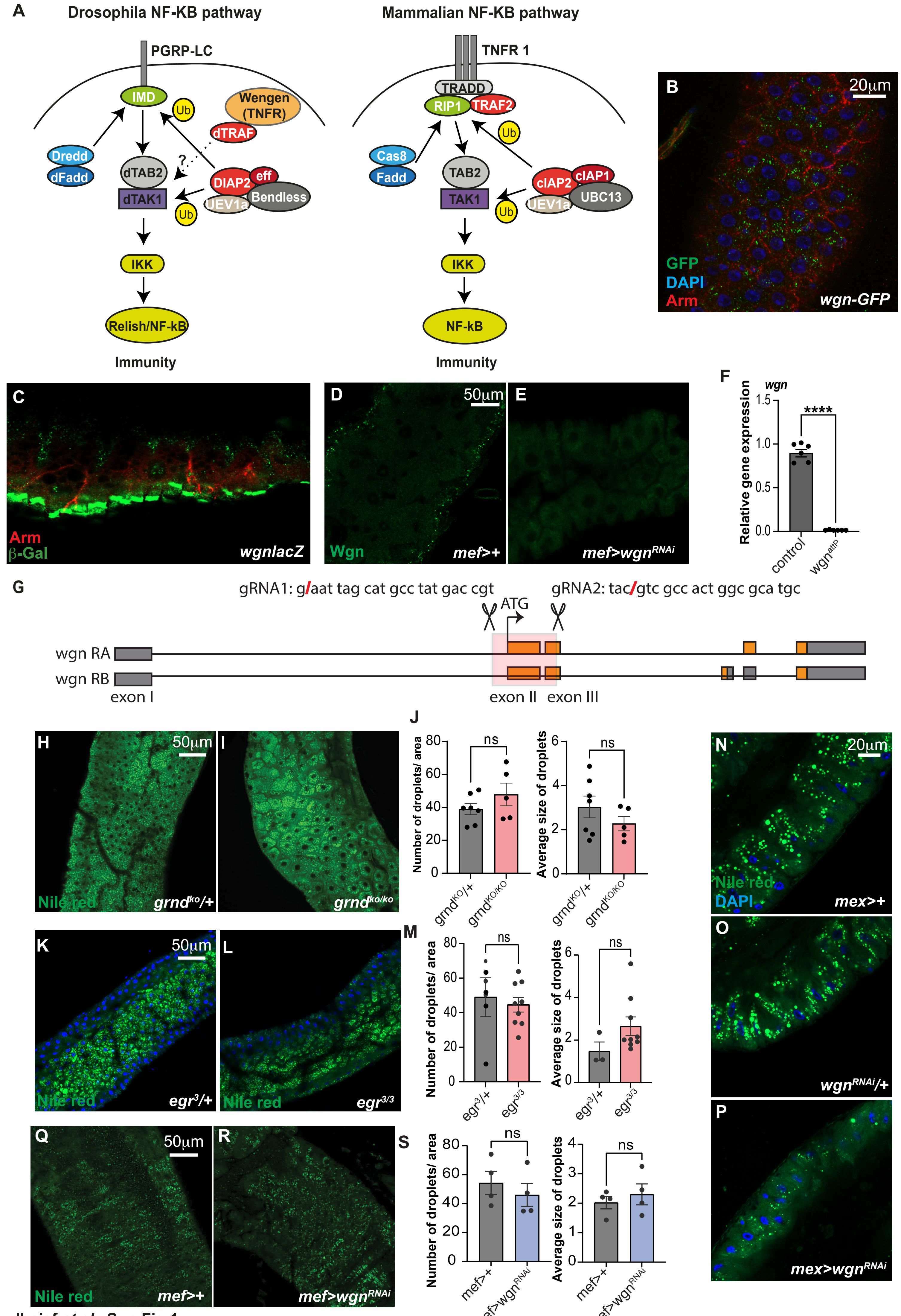

Loudhaief *et al.*, Sup Fig.1

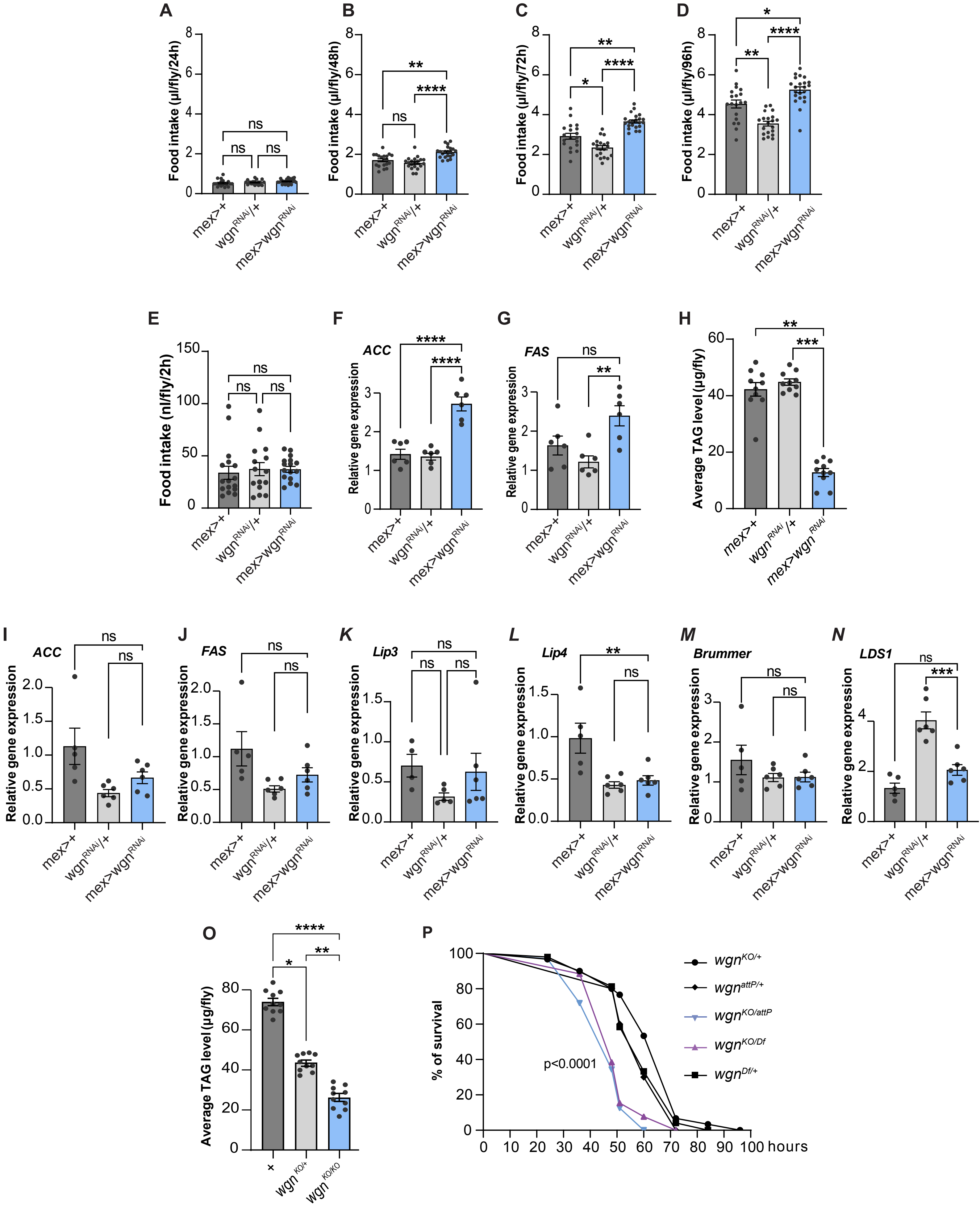

Loudhaief *et al.*, Sup Fig.2

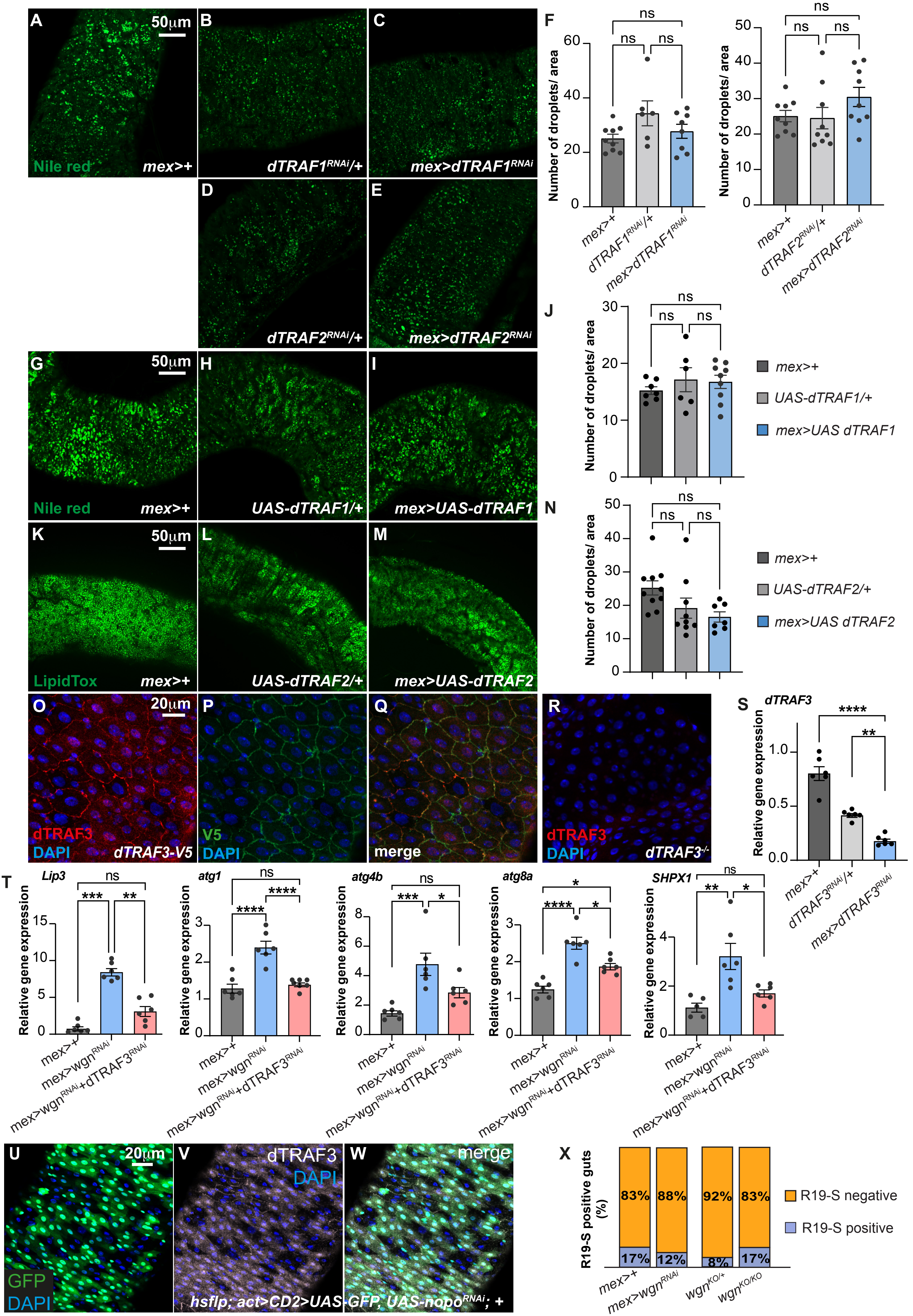
A
50μm
Nile red
mex>+
B
dTRAF1RNAi/+
C
mex>dTRAF1RNAi
D
dTRAF2RNAi/+
E
mex>dTRAF2RNAi
F
Number of droplets/ area
ns
mex>+
dTRAF1RNAi/+
mex>dTRAF1RNAi
dTRAF2RNAi/+
mex>dTRAF2RNAi
G
50μm
Nile red
mex>+
H
UAS-dTRAF1/+
I
mex>UAS-dTRAF1
J
Number of droplets/ area
ns
mex>+
UAS-dTRAF1/+
mex>UAS dTRAF1
K
50μm
LipidTox
mex>+
L
UAS-dTRAF2/+
M
mex>UAS-dTRAF2
N
Number of droplets/ area
ns
mex>+
UAS-dTRAF2/+
mex>UAS dTRAF2
O
20μm
dTRAF3
DAPI
dTRAF3-V5
P
V5
DAPI
Q
merge
R
dTRAF3
DAPI
dTRAF3-/-
S
dTRAF3
Relative gene expression
****
**
mex>+
dTRAF3RNAi/+
mex>dTRAF3RNAi
T
Lip3
atg1
atg4b
atg8a
SHPX1
Relative gene expression
ns
***
**
****
*
mex>+
mex>wgnRNAi
mex>wgnRNAi+dTRAF3RNAi
U
20μm
GFP
DAPI
V
dTRAF3
DAPI
W
merge
hsflp; act>CD2>UAS-GFP, UAS-nopoRNAi; +
X
R19-S positive guts (%)
83%
88%
92%
83%
17%
12%
8%
17%
R19-S negative
R19-S positive
mex>+
mex>wgnRNAi
wgnKO/+
wgnKO/KO

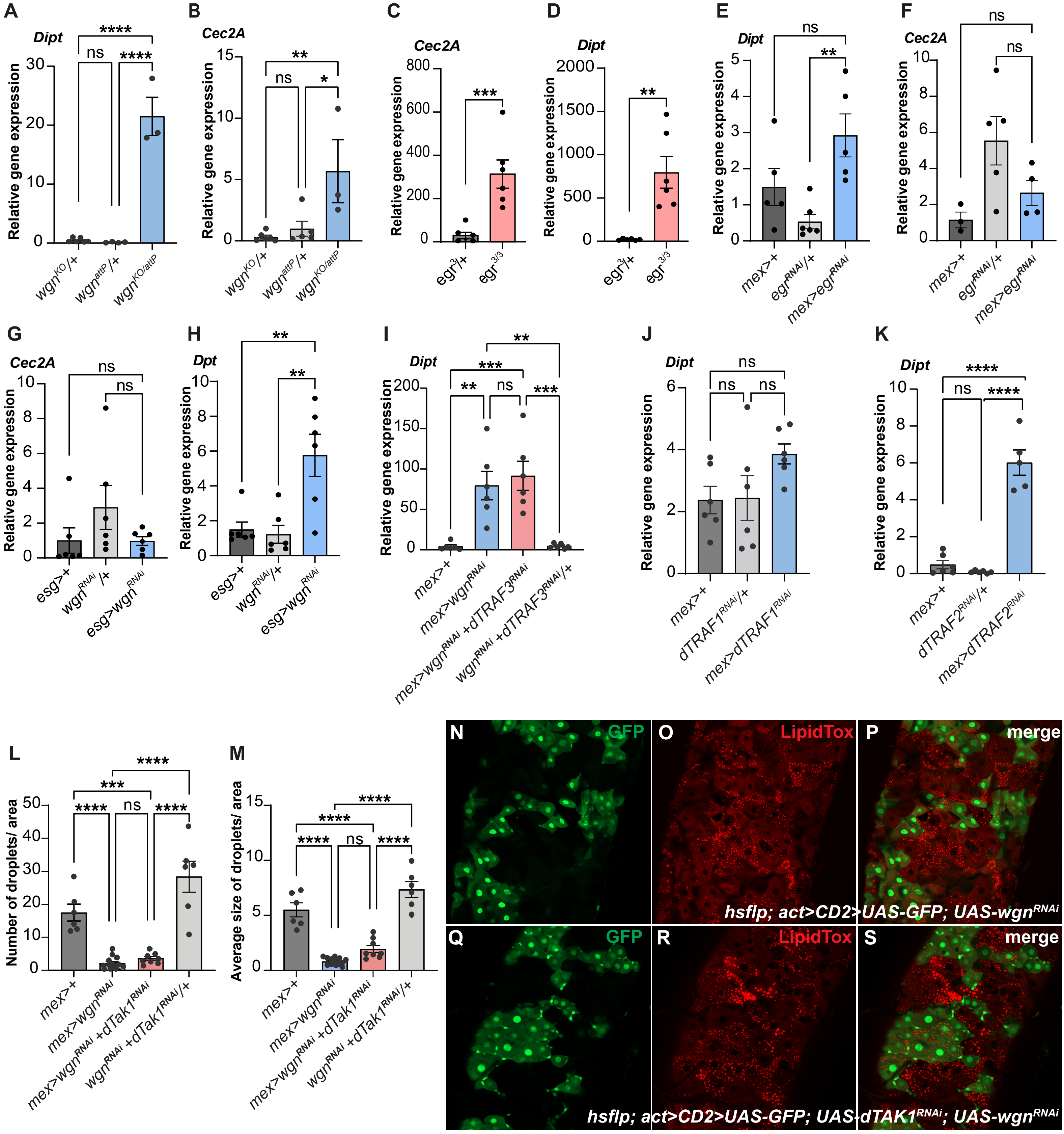

Loudhaief *et al.*, Sup Fig.4

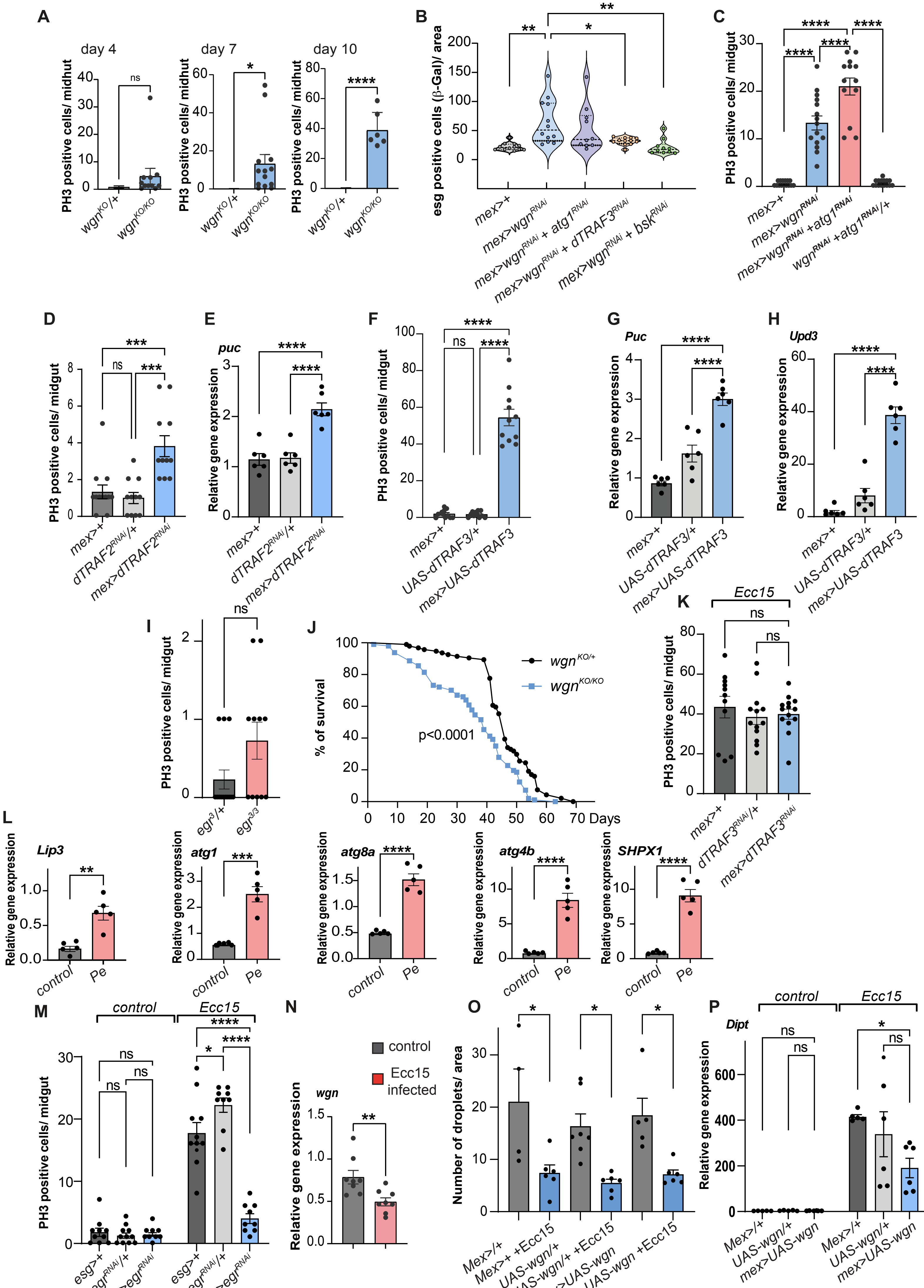

Loudhaief *et al.*, Sup Fig.5

Supplementary Figure 1: Wgn suppresses lipid catabolism independently of its ligand, Egr
(A) Schematics showing the similarities between the mammalian TNFR1/NF-κB and fly IMD/NF-κB pathways. In flies, immunity is controlled by binding of PGNs to PGRPs, which then activates IMD, a homologue of mammalian RIP1, and the downstream dTAK1-IKK-Relish/NF-kB cassette to control immunity. In mammals, binding of TNF-α to TNFR1 triggers the activation of a similar TAK1-/NF-kB cassette to promote immunity. Conserved molecules are indicated by similar colors and shapes. (B) Dissected guts from adult transgenic flies harboring endogenously GFP-tagged Wgn stained for GFP (green), Armadillo (Arm, red) and DAPI (blue) showing that Wgn is localized in cytoplasmic vesicles in ECs in the R4 region. (C) Flies expressing lacZ under the control of the *wgn* promoter stained for beta-galactosidase (β-gal) showing that Wgn is highly expressed in the VMs surrounding the gut in the R4 region. (D-E) VM-specific knockdown of Wgn showing a reduction of Wgn protein levels in the VMs surrounding the gut with RNAi-mediated depletion in R4. (F) qPCR analyses on guts dissected from control and wgn^{attp} mutant animals showing that a complete suppression of *wgn* transcript levels in wgn^{attp} mutant guts. (G) Schematic highlighting the region excised in wgn^{attp} mutants. (H-M) R2 of dissected guts from *grnd* (H-I) and *egr* (K-L) null mutant animals stained for Nile red (in green) showing that lipid stores are unaffected by loss of Grnd and Egr as quantified in J and M. (N-P) Dissected adult guts stained with Nile red (Green) and DAPI to visualize the large nuclei of ECs showing a depletion of lipids in ECs upon EC-specific depletion of Wgn in the R5 region (P). (Q-S) Nile red staining (green) of control (Q) guts and guts with VM-specific Wgn knockdown (R) showing that knockdown of Wgn in VMs does not affect number or size of lipid droplets in the R4-R5 (S).

Supplementary Figure 2: EC-specific Wgn knockdown does not reduce feeding or change the expression of genes associated with lipogenesis and lipolysis in non-intestinal tissues
(A-E) Short-term and long-term monitoring of food intake of control animals and animals with EC-specific Wgn knockdown using spectrophotometric dye-feeding and CAFÉ assays, respectively, show that food intake is slightly increased in Wgn-depleted animals. (F-G) qPCR analyses on dissected guts show that EC-specific Wgn knockdown increases ACC levels (F), while FAS levels are slightly, but not significantly, increased (G). (H) Whole-body TAG levels are reduced in animals with EC-specific Wgn depletion. (I-N) qPCR analyses on non-intestinal tissues showing that genes regulating lipogenesis (I-J) and lipolysis (K-N) are unaffected by EC-specific Wgn knockdown. (O) Whole-body TAG levels are reduced in *wgn* mutant animals. (P) Reduced resistance of trans-heterozygous *wgn* mutant flies (blue and pink) to starvation compared with control animals (grey). Error bars represent SEM; *p < 0.05, **P < 0.01, ***p < 0.001, and ****p < 0.0001.

Supplementary Figure 3: dTRAF3 is required for the induction of Lip3 and autophagy related genes associated with Wgn loss of function
(A-F) Dissected guts from control animals (A-B,D) or animals with EC-specific depletion of dTRAF1 (C) or dTRAF2 (E) stained with Nile Red (in green) show that depletion of dTRAF1 and dTRAF2 does not affect lipid stores in the R2 region as quantified in F. (G-N) Dissected guts from control animals (G-H, K-L) or animals with EC-specific ectopic expression of dTRAF1 (I) or dTRAF2 (M) stained with Nile Red show that ectopic expression of dTRAF1 and dTRAF2 does not affect lipid stores in the R2 region as quantified in J and N. (O-R) Dissected guts from flies expressing dTRAF3-V5 (green) under the control of the endogenous *dtraf3* promoter (O-Q) or *traf3* null mutant flies (R) stained with our anti-TRAF3 antibody (red) demonstrates the specificity

of the anti-TRAF3 antibody (red signal absent in R) and shows that dTRAF3 is localized at the membrane in ECs in the R5 region. (S) qPCR analyses showing that dTRAF3 levels are decreased in guts with EC-specific dTRAF3 knockdown. (T) qPCR analyses showing that the upregulation of Lip3 and genes required for autophagosome formation triggered by EC-specific Wgn depletion is suppressed by simultaneous knockdown of dTRAF3. (U-W) Adult guts carrying mosaics of wildtype (non-GFP labelled) and NOPO depleted (GFP-labelled, green) cells and stained for dTRAF3 (white, V-W) show an accumulation of cytoplasmic dTRAF3 in NOPO-depleted cells. (X) The percentage of ROS-positive guts is not increased upon global or EC-specific Wgn knockdown. Error bars represent SEM; *$p < 0.05$, **$P < 0.01$, ***$p < 0.001$, and ****$p < 0.0001$.

Supplementary Figure 4: The induction of immunity associated with Wgn loss of function does not require dTRAF3

(A-D) qPCR analyses on guts dissected from control and *wgn* and *egr* null mutant flies show that expression of Dipt and Cec2A is upregulated in *wgn* and *egr* mutant animals. (E-F) qPCR analyses on dissected guts show that Egr is not required in ECs to restrict AMP expression. (G-H) qPCR analyses on dissected guts show that knockdown of Wgn in progenitor cells has little (H) or no (G) effect on AMP expression. (I) qPCR analyses on guts of the indicated genotypes show that dTRAF3 is not required for the induction of *dipt* expression triggered by EC-specific Wgn knockdown. (J-K) qPCR analyses on guts dissected from control animals and animals with EC-specific knockdown of dTRAF1 (J) or dTRAF2 (K) shows that depletion of dTRAF2, but not dTRAF1, induces *dipt* expression. (L-M) Quantification of number of lipid droplets in the R4 region of guts dissected from the indicated genotypes shows that dTAK1 is not required for the lipid depletion associated with Wgn loss of function. (N-S) Adult guts carrying mosaics of wildtype tissue and GFP-labelled clones with Wgn (N-P) or Wgn and dTAK1 (Q-S) knockdown stained with LipidTox to label lipid droplets (in red) showing that dTAK1 is not required for the lipid depletion associated with Wgn loss of function. Error bars represent SEM; *$p < 0.05$, **$P < 0.01$, ***$p < 0.001$, and ****$p < 0.0001$.

Supplementary Figure 5: Wgn loss of function and dTRAF3 gain of function triggers proliferation in homeostatic conditions.

(A) Quantification of the number of PH3 positive cells in control and *wgn* null mutant guts dissected from animals aged 4, 7, and 10 days shows a progressive increase in the mitotic index of *wgn* mutant guts compared with control guts. (B) To monitor the mitotic index, adult guts were dissected from the indicated genotypes and the number of progenitor cells (labelled by *esg*> driven b-gal expression) per gut were counted. (C) Number of PH3-positive cells in guts with the indicated genotypes showing that Atg1 does not mediate the proliferation associated with the Wgn loss of function condition. (D-E) qPCR analyses on dissected guts showing that EC-specific dTRAF2 knockdown induces the Bsk/JNK target, *puc*, and moderately increases the number of PH3-positive cells. (F) PH3-positive cells in adult guts dissected from control animals and animals with EC-specific ectopic expression of dTRAF3. (G-H) qPCR analyses on dissected guts showing that EC-specific dTRAF3 overexpression induces *puc* (G) and *upd3* (H) expression. (I) The number of PH3-positive cells is not increased in *egr* mutant guts. (J) Survival assay shows that *wgn* mutant animals (blue) are shorter lived than control animals (black). (K) EC-specific dTRAF3 knockdown does not reduce infection-induced proliferation. (L) qPCR analyses on guts dissected from non-infected and *Pseudomonas entomophila* (P.e) infected flies showing an infection-induced upregulation of Lip3 and genes required for autophagosome formation after 16 hours of

infection. (M) Knockdown of Egr in progenitor cells suppresses the proliferative response triggered by 16 hours of *Ecc* infection. (N) qPCR analyses on dissected control (grey) or *Ecc15* infected (pink) guts show that Wgn expression is decreased after 4 hours of infection. (O) Quantification of number of lipid droplet per area in guts dissected from infected (blue bars) and non-infected (grey bars) control animals and animals with EC-specific Wgn overexpression showing that Wgn overexpression does not suppress infection-induced lipid depletion. (P) qPCR analyses on guts dissected from uninfected and infected control flies or flies with EC-specific overexpression of Wgn showing that Wgn overexpression does not reduce infection-induced Dipt expression. Error bars represent SEM; $^{*}p < 0.05$, $^{**}P < 0.01$, $^{***}p < 0.001$, and $^{****}p < 0.0001$.
